# Impact of maternal prepregnancy body mass index on neonatal outcomes following extremely preterm birth

**DOI:** 10.1002/oby.24241

**Published:** 2025-02-06

**Authors:** Charlotte Girard, Jennifer Zeitlin, Neil Marlow, Mikael Norman, Fredrik Serenius, Elizabeth S. Draper, Samantha Johnson, Valérie Benhammou, Karin Källén, Stef van Buuren, Pierre‐Yves Ancel, Andrei S. Morgan

**Affiliations:** ^1^ Obstetric, Perinatal, Paediatric and Life Course Epidemiology Team (OPPaLE), Center for Research in Epidemiology and StatisticS (CRESS) Institut National pour la Santé et la Recherche Médicale (INSERM, French Institute for Health and Medical Research), Institut National de Recherche pour l'Agriculture, l'Alimentation et l'Environnement (INRAe), Paris Cité University Paris France; ^2^ Department of Neonatology, Elizabeth Garrett Anderson Institute for Women’s Health University College London London UK; ^3^ Division of Pediatrics, Department of Clinical Science, Intervention, and Technology Stockholm Sweden; ^4^ Division of Pediatrics, Karolinska Institutet, Department of Clinical Science Intervention, and Technology Stockholm Sweden; ^5^ Department of Women's and Children's Health, Uppsala University Uppsala Sweden; ^6^ Department of Population Health Sciences, University of Leicester Leicester UK; ^7^ Center of Reproductive Epidemiology Lund University Lund Sweden; ^8^ Netherlands Organisation for Applied Scientific Research Leiden Netherlands; ^9^ Department of Methodology & Statistics Utrecht University Utrecht Netherlands; ^10^ National Maternity Hospital Dublin Ireland

## Abstract

**Objective:**

Extremes of prepregnancy maternal BMI increase neonatal mortality and morbidity at term. They also increase the risk of extremely preterm (EP, i.e., <27 weeks' gestational age) births. However, the association between maternal BMI and outcomes for EP babies is poorly understood.

**Methods:**

We used a cross‐country design, bringing together the following three population‐based, prospective, national EP birth cohorts: EXPRESS (Sweden, 2004–2007); EPICure 2 (UK, 2006); and EPIPAGE 2 (France, 2011). We included all singleton births at 22 to 26 weeks' gestational age with a live fetus at maternal hospital admission. Our exposure was maternal prepregnancy BMI, i.e., underweight, reference, overweight, or obesity. Odds ratios (OR) for survival without severe neonatal morbidity to hospital discharge according to maternal BMI were calculated using logistic regression.

**Results:**

A total of 1396 babies were born to mothers in the reference group, 140 to those with underweight, 719 to those with overweight, 556 to those with obesity, and 445 to those with missing BMI information. There was no difference in survival without major neonatal morbidity (reference, 22%; underweight, 26%, OR, 1.31, 95% CI: 0.82–2.08; overweight, 23%, OR, 1.00, 95% CI: 0.77–1.29; obesity, 19%, OR, 0.94, 95% CI: 0.70–1.25).

**Conclusions:**

No associations were seen between maternal BMI and outcomes for EP babies.

**Study Importance**

**What is already known?**

- Extremes of prepregnancy maternal BMI increase maternal, fetal, neonatal, and child mortality and morbidity among term births. They also increases the risk of extremely preterm (EP, i.e., <27 weeks' gestational age) births.
- Existing data regarding outcomes of EP babies according to maternal prepregnancy BMI provide inconsistent results. Some studies have found no links between maternal BMI and survival or neonatal outcomes, whereas others have reported higher risks of mortality and complications.

**What does the study add?**

- Using a cross‐country design with three population‐based, national, prospective EP birth cohorts that included 5273 singleton fetuses alive at maternal hospital admission, no associations were seen between prepregnancy maternal BMI and survival without major neonatal morbidity (reference, 22%; underweight, 26%; overweight, 23%; obesity, 19%).
- In secondary analyses, no associations were seen with either survival to discharge or individual neonatal morbidities.

**How might these results change the direction of research or the focus of clinical practice?**

- When counseling patients and their families about potential outcomes following EP birth, maternal prepregnancy BMI should not be considered as a factor with an important impact on survival without major morbidity.
- The focus for improving outcomes of children born to women with extremes of prepregnancy BMI should be on the prevention of EP birth, as well as trying to mitigate any consequences on longer‐term neurodevelopment for those who are born EP.

## INTRODUCTION

Overweight and obesity are major public health problems affecting all population groups, including pregnant women. Prevalence has been increasing in the United States and Europe over the past 50 years [1, 2]. In 2020, the proportion of women with obesity (body mass index [BMI] ≥ 30 kg/m^2^) of childbearing age (between ages 18 and 44 years) in the United States was estimated to be 31%, increased from 9% in 1990 [3, 4]. In Europe, in 2015, this proportion varied from 7.8% (Croatia) to 25.6% (Wales) [5]. Despite efforts to address the obesity epidemic, current predictions suggest that, by 2025, more than 21% of women in the world will have obesity [1]. Underweight women (BMI < 18.5 kg/m^2^) also constitute an important proportion of women of childbearing age, i.e., 7.5% in France and higher in low‐income countries. Notably, obesity, overweight (BMI between 25 and 30 kg/m^2^), and underweight are more prevalent among socially disadvantaged groups [2, 5, 6].

Compared with women starting pregnancy with a normal weight, women who have underweight, overweight, or obesity face increased risks of pregnancy complications and adverse outcomes. Maternal underweight is linked to nutritional deficiencies that may affect maternal well‐being and fetal growth and development [6, 7]. Clinically, this translates into higher risks for maternal death, fetal malformations, intrauterine growth restriction, and offspring malnutrition later in life [6, 7]. Increased BMI has been linked with metabolic changes, inflammation, and oxidative stress [8]. In pregnant women, these biological mechanisms affect placental structure, thereby leading to altered function and chronic hypoxia, impacting both maternal health and fetal development [9, 10]. A chronic sub‐inflammatory state increases fetal brain sensitivity to hypoxic–ischemic injury and alters brain development [10, 11]. Metabolic changes can be passed on to the fetus, upregulating growth and influencing future biological functions through epigenetic mechanisms [11]. These chronic changes may also increase the risk of mechanical difficulties at birth due to excess fetal weight [8]. Consequently, increasing BMI is associated with gestational diabetes, pregnancy‐induced hypertension, preeclampsia, instrumental delivery, cesarean section, stillbirth, macrosomia, fetal malformations, admission to a neonatal intensive care unit (NICU), and infant mortality at term [3, 8, 12, 13, 14]. Furthermore, both women with underweight and overweight or obesity show an increase in preterm birth (<37 weeks’ gestational age [GA]) [15], particularly in extremely preterm (EP, i.e., <28 weeks’ GA) birth, with a relative risk of 1.19 for underweight patients and up to 2.07 for patients with BMI ≥ 40 kg/m^2^ [16].

Although there is robust evidence that maternal BMI affects the outcomes of term infants and increases the risk of EP birth, whether it affects outcomes for EP infants is unclear. These children are more fragile than those born at term or less preterm but have reduced exposure time to the potentially deleterious effects of oxidative stress, inflammation, and metabolic changes. Existing data provide inconsistent results, and most studies only include infants admitted to NICUs.

The objective of this study was to compare survival without any major morbidity at hospital discharge of infants born EP in relation to maternal BMI in three European EP birth cohorts. By investigating this question across diverse settings, we sought to identify universal associations that may indicate biological effects as opposed to associated cultural or socioeconomic factors. We hypothesized that survival without major morbidity would be lower among EP infants of mothers with underweight, overweight, or obesity compared with infants born to mothers with normal weight. If true, this knowledge is important for discussions regarding the prognosis of these infants and for organizing their follow‐up.

## METHODS

### Data sources

We combined data from three prospectively collected, national, population‐based European cohort studies. All studies comprised births between 22 and 26 completed weeks' GA (i.e., up to 26 weeks and 6 days). The EXPRESS study included births occurring in Sweden between April 1, 2004, and March 31, 2007 (1011 births) [17]. The EPICure 2 study included births in England between January 1 and December 31, 2006 (3133 births) [18]. The EPIPAGE 2 study included births in 25 French regions: 21 of the 22 metropolitan regions and 4 overseas regions between March 28 and December 31, 2011 (2205 births) [19].

### Study population

We included all singleton fetuses alive at maternal hospital admission who were subsequently born between 22 and 26 completed weeks' GA. We excluded multiple births because outcomes may be influenced by pregnancy type. We also excluded fetuses delivered following termination of pregnancy because of differences among nations regarding late terminations (e.g., not allowed in Sweden) and practices related to detection of fetal anomalies, such as timing of the second semester ultrasound.

### Harmonization

We used variables that were previously harmonized among the three cohorts (Table S1) [20]. Additional variables were identified from the cohorts' data dictionaries and harmonized (Table S2).

### Exposure

The exposure was maternal prepregnancy BMI, derived from obstetric records (either self‐reported or measured at the first antenatal visit). Mothers were categorized into the following four groups using the standard World Health Organization (WHO) classification: underweight (<18.5 kg/m^2^); normal weight (18.5–24.9 kg/m^2^); overweight (25–29.9 kg/m^2^); or obesity (≥30 kg/m^2^).

### Outcomes

The primary outcome was survival without severe neonatal morbidity at hospital discharge. Severe neonatal morbidity was defined as the presence of at least one of the following complications: intraventricular hemorrhage grade III or IV [21]; cystic periventricular leukomalacia [20]; surgical treatment for necrotizing enterocolitis or persistent ductus arteriosus; severe bronchopulmonary dysplasia (BPD, i.e., supplemental oxygen or ventilatory support at 36 weeks' postmenstrual age) [22]; and severe retinopathy of prematurity (i.e., stage 4 or 5 of the international classification and/or treated retinopathy) [23]. Secondary outcomes were survival to hospital discharge and, among survivors, presence of any severe morbidity, as well as each morbidity individually. We also report breastfeeding status at discharge.

### Other variables

Maternal baseline characteristics included cohort of origin, age (<20 years, 20–24 years, 25–29 years, 30–34 years, and ≥35 years), presence of diabetes (either type 1 or 2) or hypertension before pregnancy, parity (nulliparous or multiparous), smoking during pregnancy (yes or no), gestational diabetes, and preeclampsia. Data on socioeconomic circumstances (e.g., education level, local deprivation indices) and maternal ethnicity or country of birth were not available for all cohorts and were therefore not included in our analyses.

Obstetric characteristics comprised antenatal transfer, antenatal steroid administration (any or none), tocolytics administration (any or none), type of preterm birth (preterm premature rupture of membrane [PPROM], spontaneous onset without PPROM, or medically indicated except PPROM), placental abruption, level of neonatal care provided at the delivery hospital (three categories), and mode of delivery (vaginal or cesarean).

Infant characteristics were GA at birth (in completed weeks), sex, birth weight *z* score (in three categories, defined using Hadlock's formula according to GA and birth weight: <−2 standard deviations [SD], between −2 and 2 SD, and >2 SD), and presence of major congenital anomalies (categorized in accordance with cohort guidelines).

### Statistical analysis

We first conducted a descriptive analysis. Maternal BMI distribution was studied in the overall population and separately in each cohort and then compared using Pearson χ^2^ test. Baseline characteristics were compared across the four BMI categories and those missing maternal BMI information. All variables were categorical and were therefore described using percentages.

**FIGURE 1:**
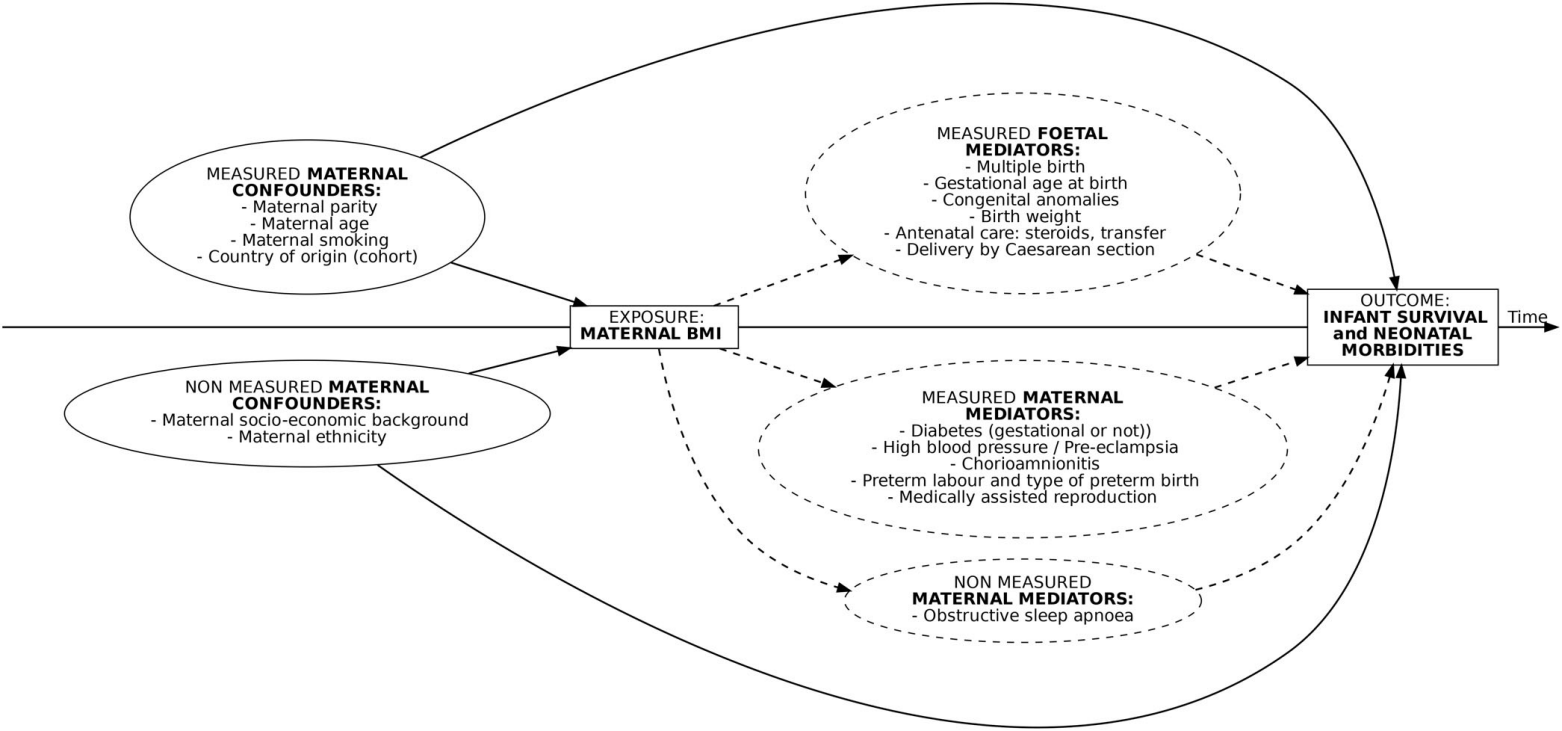
Directed acyclic graph representing the relation between maternal prepregnancy BMI and neonatal outcomes of infants born extremely preterm.

We used logistic regression to assess the relations between maternal prepregnancy BMI and the primary and secondary outcomes. Based on a literature search [3, 8, 18, 19, 24], we used a directed acyclic graph (Figure 1) to guide our models. Baseline characteristics that had been associated with both survival without neonatal morbidity in EP infants and maternal prepregnancy BMI were considered as potential confounders: maternal age; parity; smoking; and cohort of origin. Other baseline characteristics, including maternal conditions and pregnancy complications that are potentially triggered by an abnormal maternal BMI, were considered potential mediators. GA at birth can be a consequence of obesity but could also be a surrogate for factors that were not measured such as socioeconomic status, thus meaning that it might act as a confounder. It is also a known strong risk factor for neonatal morbidity. We therefore adjusted for maternal confounders and built the following two final models: without GA (considered as a mediator); and with GA (considered as a potential confounder).

Because exclusions due to missing data may lead to selection bias, all analyses were performed after imputing missing values using multiple imputation with chained equations (Table S3; details in online Supporting Information). Univariate analysis and adjusted models were run for the full sample and separately for each cohort in a stratified analysis. These models made it possible to compare outcomes among the cohorts given differences in BMI distribution and outcomes [20]. The reference group was always the normal weight group. Statistical significance was defined by a *p* value < 0.05. We report odds ratios (OR) and 95% confidence intervals (CI).

Because BMI was missing for more than 10% of the mothers, we performed a sensitivity analysis using only complete cases. We also performed a hierarchical analysis including multiple pregnancies to account for the shared environment of these babies using complete cases.

All analyses were performed in R (version 4.1.2, R Project for Statistical Computing) and reported according to the Strengthening the Reporting of Observational Studies in Epidemiology (STROBE) guidelines (Table S4).

### Ethics

All three cohorts were approved by national ethics committees. Permission for transfer and use of the anonymized datasets was provided by the Regional Research Ethics Board (Lund University, Lund, Sweden) for the EXPRESS data and by their respective data owners and study sponsors for the EPICure 2 data and the EPIPAGE 2 data.

## RESULTS

### Descriptive analysis

**FIGURE 2:**
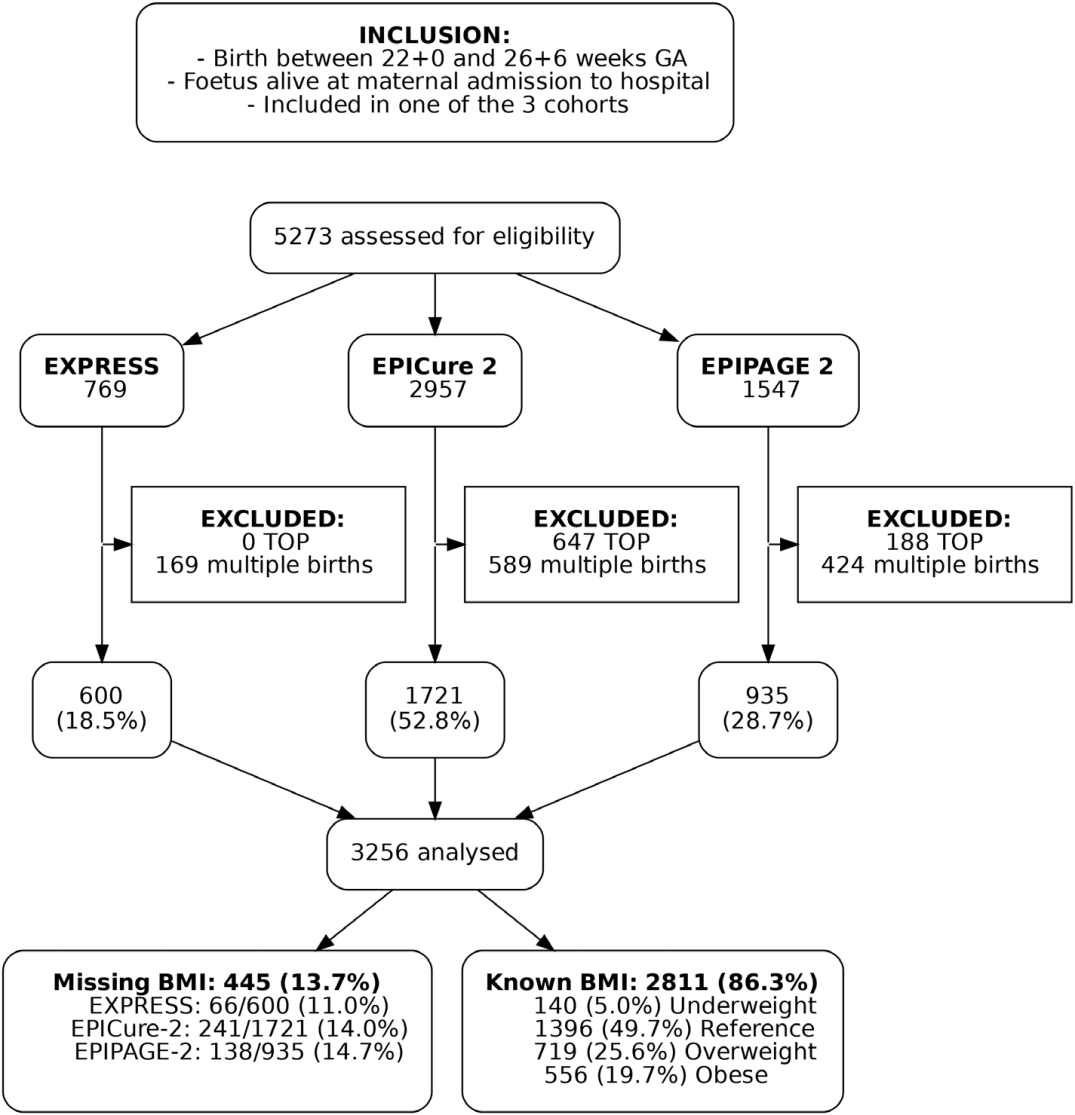
Flowchart of the study population. GA, gestational age; TOP, termination of pregnancy.

**FIGURE 3:**
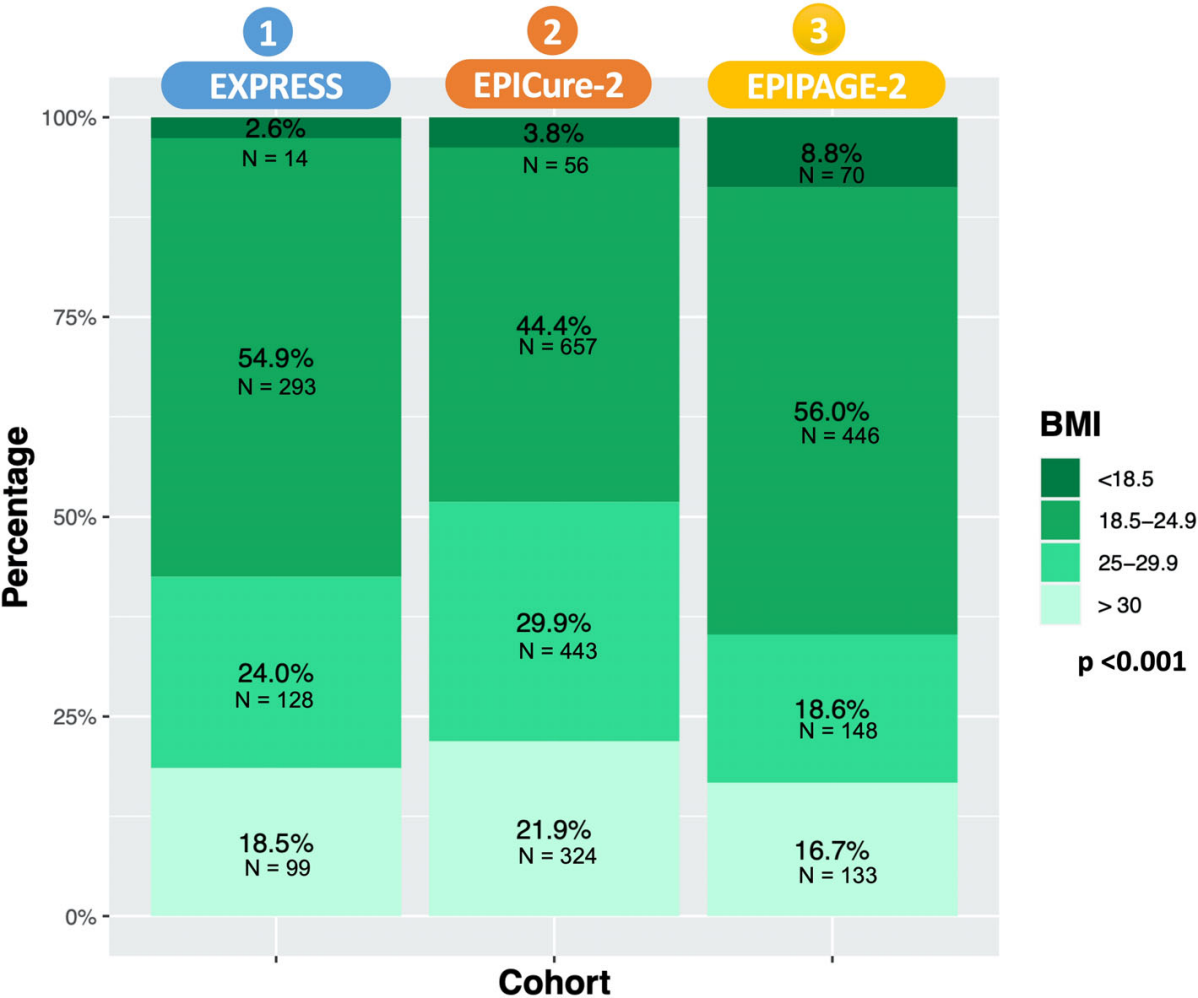
BMI distribution among the three cohorts. [Color figure can be viewed at wileyonlinelibrary.com]

A total of 5273 fetuses were alive at maternal hospital admission and subsequently born between 22 and 26 completed weeks’ GA in the three cohorts (Figure 2). After exclusions, 2811 (86.3%) of the 3256 fetuses had known maternal prepregnancy BMI: 1396 (49.7%) were born to mothers in the reference group; 140 (5.0%) were born to mothers with underweight; 719 (25.6%) were born to mothers with overweight; and 556 (19.7%) were born to mothers with obesity (Figure 2). The BMI distributions were different among the three cohorts (*p* < 0.001; Figure 3). EPIPAGE 2 included 70 (8.8%) fetuses who were born to mothers with underweight, whereas EXPRESS included only 14 (2.6%). Over one‐half of the mothers in EPICure 2 had overweight (29.9%) or obesity (21.9%).

When comparing baseline characteristics between participants with and without maternal BMI available, no major differences were seen for maternal characteristics, but fetuses with missing maternal BMI were less likely to have received corticosteroids antenatally and to have a congenital malformation and were more likely to have been born in a level 1 hospital (Table 1). Maternal baseline characteristics differed among the four BMI groups (Table 1). Women with underweight were younger and smoked more. Mothers who had overweight or obesity were older and were more likely to present with prepregnancy diabetes, prepregnancy high blood pressure, gestational diabetes, and preeclampsia. When stratifying by cohort (Tables S5–S7), mothers who had overweight or obesity were more likely than the reference group to be primigravidas in EPICure 2 (52.6% and 52.7%, respectively), but not in EXPRESS (38.3% and 32.3%, respectively) or EPIPAGE 2 (49.3% and 42.5%, respectively). In EXPRESS, mothers with obesity were more frequently smokers (18.0%) than those in the reference group (8.6%), which was not seen in the other two cohorts (15.3% and 30.1% in EPICure 2 and 15.5% and 24.2% in EPIPAGE 2, respectively). Birth characteristics were also compared according to the BMI groups (Table 1). Women with underweight were more likely to have received antenatal tocolytics and to be treated for PPROM. Mothers who had overweight or obesity were more likely to deliver for medical reasons. Infant characteristics, including GA at birth or birth weight, did not differ according to maternal BMI (Table 1). There were few differences in neonatal outcomes by maternal prepregnancy BMI group (Table 2), although infants born to mothers with obesity were less likely to be breastfed at discharge.

**TABLE 1:** Baseline characteristics according to BMI of mothers with singleton pregnancies whose fetuses were alive at maternal hospital admission, from three prospective national cohort studies conducted in Sweden (EXPRESS, 2004–2007), England (EPICure 2, 2006), and France (EPIPAGE 2, 2011).

	Complete cases, *n* = 2811				Missing BMI, *n* = 445	Total, = 3256
	BMI < 18.5, *n* = 140	BMI 18.5–24.9, *n* = 1396	BMI 25–29.9, *n* = 719	BMI > 30, *n* = 556		
*Mother*						
Maternal age, y						
<20	20/140 (14.3)	123/1393 (8.8)	33/718 (4.6)	14/555 (2.5)	38/440 (8.6)	228/3246 (7.0)
20–24	43/140 (30.7)	266/1393 (19.1)	128/718 (17.8)	83/555 (15.0)	89/440 (20.2)	609/3246 (18.8)
25–29	41/140 (29.3)	399/1393 (28.6)	206/718 (28.7)	152/555 (27.4)	118/440 (26.8)	916/3246 (28.2)
30–34	27/140 (19.3)	332/1393 (23.8)	178/718 (24.8)	156/555 (28.1)	103/440 (23.4)	796/3246 (24.5)
35+	9/140 (6.4)	273/1393 (19.6)	173/718 (24.1)	150/555 (27.0)	92/440 (20.9)	697/3246 (21.5)
Nulliparity	60/139 (43.2)	628/1388 (45.2)	353/715 (49.4)	254/548 (46.4)	206/435 (47.4)	1501/3225 (46.5)
Smoking	43/134 (32.1)	319/1352 (23.6)	166/692 (23.9)	86/540 (15.9)	72/338 (21.3)	686/3056 (22.4)
Prepregnancy diabetes	0/137 (0.0)	8/1370 (0.6)	6/713 (0.8)	17/552 (3.1)	4/427 (0.9)	35/3199 (1.1)
Gestational diabetes	1/132 (0.8)	8/1334 (0.6)	10/697 (1.4)	18/542 (3.3)	5/412 (1.2)	42/3117 (1.3)
Prepregnancy high BP	2/140 (1.4)	21/1396 (1.5)	23/719 (3.2)	47/556 (8.5)	12/445 (2.7)	105/3256 (3.2)
Preeclampsia	4/140 (2.9)	101/1396 (7.2)	67/719 (9.3)	62/556 (11.2)	39/445 (8.8)	273/3256 (8.4)
*Obstetrics*						
Antenatal transfer	44/137 (32.1)	439/1362 (32.2)	203/708 (28.7)	159/550 (28.9)	134/434 (30.9)	979/3191 (30.7)
Antenatal steroids	86/134 (64.2)	942/1367 (68.9)	503/709 (70.9)	371/547 (67.8)	252/413 (61.0)	2154/3170 (67.9)
Antenatal tocolytics	63/136 (46.3)	571/1376 (41.5)	251/710 (35.4)	198/551 (35.9)	154/429 (35.9)	1237/3202 (38.6)
Type of prematurity						
PPROM	47/132 (35.6)	411/1319 (31.2)	199/696 (28.6)	135/534 (25.3)	113/408 (27.7)	905/3089 (29.3)
Spontaneous	71/132 (53.8)	727/1319 (55.1)	394/696 (56.6)	300/534 (56.2)	227/408 (55.6)	1719/3089 (55.6)
Medically indicated	14/132 (10.6)	181/1319 (13.7)	103/696 (14.8)	99/534 (18.5)	68/408 (16.7)	465/3089 (15.1)
Placental abruption	11/136 (8.1)	132/1365 (9.7)	54/710 (7.6)	38/548 (6.9)	25/423 (5.9)	260/3182 (8.2)
Level of birth hospital						
1	10/137 (7.3)	95/1382 (6.9)	54/710 (7.6)	41/555 (7.4)	26/439 (5.9)	226/3223 (7.0)
2	29/137 (21.2)	393/1382 (28.4)	203/710 (28.6)	144/555 (25.9)	136/439 (31.0)	905/3223 (28.1)
3	98/137 (71.5)	894/1382 (64.7)	453/710 (63.8)	370/555 (66.7)	277/439 (63.1)	2092/3223 (64.9)
Mode of delivery						
Vaginal	99/137 (72.3)	1002/1385 (72.3)	537/719 (74.7)	383/551 (69.5)	312/436 (71.6)	2333/3228 (72.3)
Cesarean section	38/137 (27.7)	383/1385 (27.7)	182/719 (25.3)	168/551 (30.5)	124/436 (28.4)	895/3228 (27.7)
*Infant*						
GA, wk						
22	18/140 (12.9)	146/1396 (10.5)	85/719 (11.8)	79/556 (14.2)	53/445 (11.9)	381/3256 (11.7)
23	18/140 (12.9)	218/1396 (15.6)	104/719 (14.5)	90/556 (16.2)	84/445 (18.9)	514/3256 (15.8)
24	25/140 (17.9)	287/1396 (20.6)	152/719 (21.1)	112/556 (20.1)	81/445 (18.2)	657/3256 (20.2)
25	42/140 (30.0)	354/1396 (25.4)	166/719 (23.1)	135/556 (24.3)	107/445 (24.0)	804/3256 (24.7)
26	37/140 (26.4)	391/1396 (28.0)	212/719 (29.5)	140/556 (25.2)	120/445 (27.0)	900/3256 (27.6)
Sex, female	73/140 (52.1)	617/1392 (44.3)	345/719 (48.0)	259/555 (46.7)	198/443 (44.7)	1492/3249 (45.9)
Birth weight *z* score^a^						
<−2 SD	15/137 (10.9)	137/1379 (9.9)	90/715 (12.6)	73/550 (13.3)	46/436 (10.6)	361/3217 (11.2)
Between −2 and 2 SD	120/137 (87.6)	1225/1379 (88.8)	613/715 (85.7)	469/550 (85.3)	383/436 (87.8)	2810/3217 (87.3)
>2 SD	2/137 (1.5)	17/1379 (1.2)	12/715 (1.7)	8/550 (1.5)	7/436 (1.6)	46/3217 (1.4)
Congenital anomalies	2/139 (1.4)	56/1375 (4.1)	21/702 (3.0)	23/549 (4.2)	11/432 (2.5)	113/3197 (3.5)

*Note*: Data given as *n*/*N* (%).

Abbreviations: BP, blood pressure; GA, gestational age; PPROM, preterm premature rupture of membranes.

^a^ Calculated with Hadlock's formula: *z* score = (birth weight − Mean)/SD, where mean = exp(0.578 + 0.332 × GA − 0.00354 × GA^2^) and SD = 0.127 × (exp[0.578 + 0.332 × GA − 0.00354 × GA^2^]).

**TABLE 2:** Descriptive analysis of outcomes according to BMI of mothers with singleton pregnancies whose fetuses were alive at maternal hospital admission, from three prospective national cohort studies conducted in Sweden (EXPRESS, 2004–2007), England (EPICure 2, 2006), and France (EPIPAGE 2, 2011)

	Complete cases				Missing BMI	Total
	BMI < 18.5	BMI 18.5–24.9	BMI 25–29.9	BMI > 30		
*Fetuses alive at maternal hospital admission*	*n* = 140	*n* = 1396	*n* = 719	*n* = 556	*n* = 445	*n* = 3256
Survival at discharge without any severe morbidity^a^	36/140 (25.7)	309/1396 (22.1)	163/719 (22.7)	108/556 (19.4)	88/445 (20)	704/3256 (21.6)
Survival at discharge	65/140 (46.4)	677/1396 (48.5)	361/719 (50.2)	255/556 (45.9)	204/445 (45.8)	1562/3256 (48.0)
*Among survivors at discharge*	*n* = 65	*n* = 677	*n* = 361	*n* = 255	*n* = 204	*n* = 1562
Any severe morbidity^a^	29/65 (44.6)	368/677 (54.4)	198/361 (54.8)	147/255 (57.6)	116/204 (56.9)	858/1562 (54.9)
IVH						
0	34/65 (52.3)	338/674 (50.1)	191/359 (53.2)	134/254 (52.8)	108/203 (53)	805/1555 (51.8)
1	5/65 (7.7)	126/674 (18.7)	64/359 (17.8)	41/254 (16.1)	32/203 (15.8)	268/1555 (17.2)
2	14/65 (21.5)	120/674 (17.8)	55/359 (15.3)	49/254 (19.3)	32/203 (15.8)	270/1555 (17.4)
3	6/65 (9.2)	34/674 (5.0)	20/359 (5.6)	11/254 (4.3)	14/203 (6.9)	85/1555 (5.5)
4	6/65 (9.2)	56/674 (8.3)	29/359 (8.1)	19/254 (7.5)	17/203 (8.4)	127/1555 (8.2)
cPVL	4/65 (6.2)	29/676 (4.3)	18/359 (5.0)	14/254 (5.5)	12/204 (5.9)	77/1558 (4.9)
NEC: surgically treated	3/65 (4.6)	38/676 (5.6)	17/361 (4.7)	12/255 (4.7)	16/200 (8.0)	86/1557 (5.5)
PDA: surgically treated	10/64 (15.6)	148/669 (22.1)	70/357 (19.6)	48/249 (19.3)	37/202 (18.3)	313/1541 (20.3)
BPD						
None/mild	32/61 (52.5)	270/642 (42.1)	123/342 (36.0)	84/245 (34.3)	79/196 (40.3)	588/1486 (39.6)
Moderate	13/61 (21.3)	180/642 (28.0)	106/342 (31.0)	68/245 (27.8)	53/196 (27.0)	420/1486 (28.3)
Severe	16/61 (26.2)	192/642 (29.9)	113/342 (33.0)	93/245 (38.0)	64/196 (32.7)	478/1486 (32.2)
ROP (stages)						
0	28/54 (51.9)	227/602 (37.7)	130/335 (38.8)	87/242 (36.0)	68/185 (36.8)	540/1418 (38.1)
1	7/54 (13.0)	102/602 (16.9)	62/335 (18.5)	45/242 (18.6)	45/185 (24.3)	261/1418 (18.4)
2	13/54 (24.1)	140/602 (23.3)	73/335 (21.8)	60/242 (24.8)	31/185 (16.8)	317/1418 (22.4)
3	6/54 (11.1)	129/602 (21.4)	67/335 (20.0)	50/242 (20.7)	39/185 (21.1)	291/1418 (20.5)
4	0/54 (0.0)	2/602 (0.3)	2/335 (0.6)	0/242 (0.0)	1/185 (0.5)	5/1418 (0.4)
5	0/54 (0.0)	2/602 (0.3)	1/335 (0.3)	0/242 (0.0)	1/185 (0.5)	4/1418 (0.3)
ROP (treated)	4/26 (15.4)	97/393 (24.7)	54/207 (26.1)	36/153 (23.5)	26/112 (23.2)	217/891 (23.2)
Breastfeeding at discharge	24/56 (42.9)	312/641 (48.7)	158/354 (44.6)	91/241 (37.8)	72/195 (36.9)	657/1487 (36.9)

*Note*: Data given as *n*/*N* (%).

Abbreviations: BPD, bronchopulmonary dysplasia; cPVL, cystic periventricular leukomalacia; IVH, intraventricular hemorrhage; NEC, necrotizing enterocolitis; PDA, persistent ductus arteriosus; ROP, retinopathy of prematurity.

^a^ Severe neonatal morbidity: severe neurological injury (severe IVH grade III or IV using the Papile et al. [25] classification and/or cPVL according to de Vries et al. [26]), surgical treatment for NEC, surgical treatment for PDA, severe BPD (use of supplemental oxygen at 36 weeks' postmenstrual age), and severe ROP (stage 4 or 5 of the international classification and/or treated).

### Primary outcome

The univariate analysis did not find any differences in survival without severe morbidity at discharge among the reference group and the mothers with underweight (OR, 1.22, 95% CI: 0.81–1.80), overweight (OR, 1.03, 95% CI: 0.83–1.28), or obesity (OR, 0.85, 95% CI: 0.66–1.08). These results did not vary when adjusting for cohort and potential confounders (i.e., maternal age, parity, and smoking) or when also adjusting for GA (underweight, OR, 1.40, 95% CI: 0.88–2.19; overweight, OR, 1.07, 95% CI: 0.84–1.37; obesity, OR, 0.94, 95% CI: 0.71–1.24). No differences were seen when using complete cases in the univariate analysis or after adjustment for cohort, potential confounders, and GA. Full results are shown in Table 3; the sensitivity analysis including multiple births yielded consistent results (data available on request).

**TABLE 3:** Primary outcome of survival without any severe neonatal morbidity^a^ of fetuses alive at maternal hospital admission, from three prospective national cohort studies conducted in Sweden (EXPRESS, 2004–2007), England (EPICure 2, 2006), and France (EPIPAGE 2, 2011), according to BMI of mothers.

	Univariate analysis		With adjustment for maternal confounders^b^ and cohort (when not stratified)		With adjustment for maternal confounders^b^, cohort (when not stratified), and GA at birth	
BMI	OR	95% CI	aOR	95% CI	aOR	95% CI
Imputed data, *n* = 3256						
18.5–24.9	Ref		Ref		Ref	
<18.5	1.21	0.80–1.81	1.27	0.83–1.92	1.31	0.82–2.08
25–29.9	1.00	0.80–1.26	1.03	0.82–1.30	1.00	0.77–1.29
≥30	0.87	0.67–1.12	0.89	0.68–1.15	0.94	0.70–1.25
Complete cases, *n* = 2811			*n* = 2693		*n* = 2693	
18.5–24.9	Ref		Ref		Ref	
<18.5	1.22	0.81–1.80	1.34	0.88–2.01	1.40	0.88–2.19
25–29.9	1.03	0.83–1.28	1.10	0.87–1.37	1.07	0.84–1.37
≥30	0.85	0.66–1.08	0.90	0.69–1.15	0.94	0.71–1.24
Imputed data: EXPRESS, *n* = 600						
18.5–24.9	Ref		Ref		Ref	
<18.5	3.41	1.12–10.04	3.27	1.04–10.20	2.94	0.86–10.10
25–29.9	1.41	0.87–2.29	1.33	0.81–2.19	1.31	0.73–2.36
≥30	1.18	0.69–1.99	1.14	0.66–1.98	1.14	0.62–2.11
Imputed data: EPICure 2, *n* = 1721						
18.5–24.9	Ref		Ref		Ref	
<18.5	0.79	0.37–1.68	0.78	0.36–1.67	0.85	0.37–1.91
25–29.9	0.97	0.70–1.34	0.96	0.69–1.32	0.86	0.61–1.23
≥30	0.96	0.67–1.37	0.92	0.64–1.32	0.97	0.65–1.43
Imputed data: EPIPAGE 2, *n* = 935						
18.5–24.9	Ref		Ref		Ref	
<18.5	1.22	0.69–2.17	1.30	0.72–2.34	1.35	0.69–2.64
25–29.9	0.96	0.61–1.50	0.92	0.59–1.45	1.06	0.64–1.76
≥30	0.60	0.36–1.03	0.60	0.35–1.03	0.67	0.37–1.22

Abbreviations: aOR, adjusted odds ratio; GA, gestational age; OR, odds ratio; Ref, reference group.

^a^ Severe neonatal morbidity: severe neurological injury (severe intraventricular hemorrhage grade III or IV using the Papile et al [25]. classification and/or cystic periventricular leukomalacia according to de Vries et al. [26]), surgical treatment for necrotizing enterocolitis, surgical treatment for persistent ductus arteriosus, severe bronchopulmonary dysplasia (use of supplemental oxygen at 36 weeks' postmenstrual age), and severe retinopathy of prematurity (stage 4 or 5 of the international classification and/or treated).

^b^ Maternal confounders were age, parity, and smoking.

When stratifying by cohort, the mothers with underweight in EXPRESS had higher survival without morbidity than the reference group in the univariate analysis and after adjustment for maternal confounders (OR, 3.27, 95% CI: 1.12–10.04). There were no differences for the mothers with overweight (OR, 1.31, 95% CI: 0.73–2.36) or obesity (OR, 1.14, 95% CI: 0.62–2.11). In EPICure 2, estimates were similar across groups (underweight, OR, 0.79, 95% CI: 0.37–1.68; overweight, OR, 0.97, 95% CI: 0.79–1.34; obesity, OR, 0.96, 95% CI: 0.67–1.37). In EPIPAGE 2, the mothers with obesity had lower survival without severe morbidity when compared with the reference group, but CI included one (OR, 0.60, 95% CI: 0.35–1.03) when adjusted for maternal confounders. Full results by cohort are shown in Tables S8–S10.

### Secondary outcomes

There were no statistically significant differences seen among maternal BMI groups aside from a higher risk of severe BPD in the mothers with obesity (OR, 1.42, 95% CI: 1.05–1.93) and a lower risk of severe retinopathy of prematurity in the mothers with underweight (OR, 0.34, 95% CI: 0.12–0.95). However, these differences were only observed in the unadjusted analysis, not after adjustment for potential confounders (Table 4).

**TABLE 4:** Secondary outcomes using imputed data (*n* = 3256) for fetuses alive at maternal hospital admission, from three prospective national cohort studies conducted in Sweden (EXPRESS, 2004–2007), England (EPICure 2, 2006), and France (EPIPAGE 2, 2011), according to BMI of mothers.

	Univariate analysis		With adjustment for maternal confounders^a^ and cohort		With adjustment for maternal confounders^a^, cohort, and GA at birth	
BMI	OR	95% CI	aOR	95% CI	aOR	95% CI
Survival at discharge						
18.5–24.9	Ref		Ref		Ref	
<18.5	0.91	0.64–1.28	1.08	0.75–1.54	1.01	0.70–1.46
25–29.9	1.07	0.90–1.28	1.05	0.88–1.26	1.05	0.87–1.27
≥30	0.92	0.76–1.12	0.90	0.73–1.10	0.97	0.79–1.20
At least one severe morbidity^b^ at discharge^c^						
18.5–24.9	Ref		Ref		Ref	
<18.5	0.68	0.41–1.15	0.74	0.43–1.26	0.75	0.43–1.29
25–29.9	1.06	0.81–1.39	1.00	0.76–1.32	1.03	0.77–1.38
≥30	1.13	0.84–1.52	1.05	0.78–1.43	1.04	0.76–1.43
Severe intraventricular hemorrhage: grade III or IV^c^						
18.5–24.9	Ref		Ref		Ref	
<18.5	1.37	0.70–2.69	1.58	0.79–3.17	1.61	0.80–3.23
25–29.9	1.00	0.69–1.45	0.86	0.59–1.26	0.87	0.59–1.28
≥30	0.86	0.55–1.32	0.73	0.46–1.13	0.72	0.46–1.13
Cystic periventricular leukomalacia^c^						
18.5–24.9	Ref		Ref		Ref	
<18.5	1.42	0.49–4.10	1.59	0.54–4.70	1.60	0.54–4.75
25–29.9	1.14	0.63–2.08	1.04	0.56–1.91	1.04	0.57–1.92
≥30	1.26	0.65–2.44	1.23	0.63–2.42	1.23	0.63–2.41
Severe necrotizing enterocolitis: surgically treated^c^						
18.5–24.9	Ref		Ref		Ref	
<18.5	0.80	0.24–2.67	0.76	0.22–2.57	0.76	0.23–2.60
25–29.9	0.92	0.52–1.64	0.88	0.49–1.60	0.89	0.49–1.60
≥30	0.89	0.45–1.73	0.88	0.44–1.73	0.87	0.44–1.71
Severe persistent ductus arteriosus: surgically treated^c^						
18.5–24.9	Ref		Ref		Ref	
<18.5	0.71	0.36–1.39	0.75	0.75–1.49	0.77	0.38–1.57
25–29.9	0.87	0.64–1.19	0.95	0.95–1.30	0.96	0.69–1.34
≥30	0.84	0.59–1.20	0.88	0.88–1.27	0.85	0.58–1.25
Severe bronchopulmonary dysplasia^c^						
18.5–24.9	Ref		Ref		Ref	
<18.5	0.84	0.47–1.52	0.87	0.48–1.60	0.89	0.48–1.64
25–29.9	1.16	0.88–1.53	1.07	0.80–1.43	1.10	0.82–1.47
≥30	1.42	1.05–1.93	1.31	0.96–1.80	1.30	0.94–1.80
Severe retinopathy of prematurity: stage 4 or 5 and/or treated^c^						
18.5–24.9	Ref		Ref		Ref	
<18.5	0.34	0.12–0.95	0.44	0.15 1.27	0.47	0.16–1.37
25–29.9	1.07	0.74–1.55	0.99	0.68–1.46	1.04	0.70–1.55
≥30	1.04	0.68–1.61	0.95	0.61–1.51	0.96	0.60–1.54

Abbreviations: aOR, adjusted odds ratio; GA, gestational age; OR, odds ratio; Ref, reference group.

^a^ Maternal confounders include age, parity, and smoking.

^b^ Severe neonatal morbidity: severe neurological injury (severe intraventricular hemorrhage grade III or IV using the Papile et al [25]. classification and/or cystic periventricular leukomalacia according to de Vries et al. [26]), surgical treatment for necrotizing enterocolitis, surgical treatment for persistent ductus arteriosus, severe bronchopulmonary dysplasia (use of supplemental oxygen at 36 weeks' postmenstrual age), and severe retinopathy of prematurity (stage 4 or 5 of the international classification and/or treated).

^c^ Among survivors at discharge.

## DISCUSSION

### Principal findings

Among infants born before 27 weeks’ GA included in these three European prospectively collected national cohorts, there were no consistent associations between maternal prepregnancy BMI and survival to discharge without severe neonatal morbidity or between maternal prepregnancy BMI and survival to discharge or any of the individual neonatal morbidities.

### Strength and limitations

This study included high‐quality data from three large, national, prospective EP birth cohorts. The diversity of the patient populations and the settings was a strength, as we hypothesized a biological association between maternal BMI and neonatal outcomes that we expected to observe in all contexts [8, 11]. We were able to include all fetuses alive at hospital admission as opposed to only live births or babies admitted to NICUs. This is important for comparative research given the highly variable proportions of intrapartum and labor ward deaths that occur at these gestations [20].

Limitations include the age of the data that were collected over 10 years ago, as standards of care have changed in the interim. However, the pathophysiological mechanisms underlying our hypotheses would not be affected by this. A further limitation is missing maternal BMI for more than 10% of infants. We imputed missing data, and results between imputed and complete case analyses were consistent. In our study, due to harmonization difficulties, we were unable to adjust for ethnicity or socioeconomic background, but including these factors would most likely reduce differences further among the BMI groups [2, 6, 8]. Moreover, the impact of social factors on EP neonatal outcomes is not pronounced [27], and this lack of adjustment is unlikely to obscure an existing biological impact. Finally, we made multiple comparisons among a large number of secondary outcomes, and results should therefore be interpreted cautiously.

### Comparison with literature

Some studies have reported higher risks of resuscitation in the delivery room [28], mortality [29], severe asphyxia‐related complications [30], intraventricular hemorrhage [31], and BPD [32]. On the other hand, two studies of maternal BMI and EP neonatal outcomes found no links between maternal BMI and survival [33] or neonatal outcomes [34], and many other studies have had consistency with our results in finding no association [29, 34, 35]. Extremes of BMI may have more impact on neonatal outcomes of later preterm or term infants [3, 8] because they are exposed to adverse biological conditions for longer. For example, a critical part of fetal brain development occurs in the third trimester, by which point EP children have already been born. Furthermore, the strong impact of EP birth itself on mortality and morbidity risks [18, 19, 24] may obscure less‐pronounced vulnerabilities due to maternal BMI. Additionally, studies on 10‐year outcomes for EP infants have shown altered neurological development in those born to mothers with obesity [36, 37]. However, because we found no link between maternal obesity and neonatal morbidity and mortality at hospital discharge for EP infants, the poor long‐term neurodevelopment of these neonates may be associated with factors that do not affect their immediate development and survival.

### Implications

In the context of EP birth, children are at very high risk of mortality and morbidity [38]. Active management and intensity of care are usually discussed before and after the birth by the medical team and with the parents [39]. An understanding of prognostic factors is central to informed decision‐making. Our results provide further confirmation that maternal BMI should not weigh in the balance when considering neonatal outcomes of children born EP. We did note lower survival and morbidity in infants born to mothers with obesity in France, which was not seen in the other cohorts. This raises the possibility that clinical beliefs, i.e., that maternal BMI has a deleterious effect, may affect active management decisions in some settings. Further research exploring management of births after 26 weeks in the French cohort according to maternal BMI could shed more light on this hypothesis.

Studies investigating maternal BMI and longer‐term EP outcomes are scarce but have consistently reported worse neurodevelopmental outcomes in children born to mothers with obesity [36, 37], which is also the case for children born at term [8, 14]. It is necessary to understand when and why such differences appear in childhood to allow for prevention and early intervention. Finally, although maternal BMI and, in particular, obesity did not affect neonatal outcomes among EP infants, overweight and obesity are linked to higher rates of EP birth [15, 16], and this risk should be taken into account when caring for such patients.

## CONCLUSION

In this large, prospectively collected, multinational, population‐based European cohort study, we did not find any association between maternal prepregnancy BMI and survival without severe morbidity among infants born EP, in contrast with findings in infants born at term. In order to improve outcomes of children born to women with extreme prepregnancy BMI, public health policies should focus on preventing EP birth and mitigating the adverse consequences of EP birth for longer‐term neurodevelopment.

## FUNDING INFORMATION

Charlotte Girard was supported by funding from the Société Française de Médecine Périnatale (French Society of Perinatal Medicine) and the Ministry for Health, French. The EXPRESS cohort study was supported by the Swedish Research Council grants 2006–3855 and 2009–4250, the Uppsala‐Örebro Regional Research Council grant RFR‐10324, a grant from the Research Council South East Region of Sweden, and grants to Researchers in the Public Health Care from the Swedish government. Financial support was also provided through a regional agreement between the University of Umeå and Västerbotten County Council and through a regional agreement on medical training and clinical research (ALF) between Stockholm County Council and Karolinska Institutet. The study also received support from The “Lilla Barnets Fond” Children's fund, the Evy and Gunnar Sandberg and the Birgit and Håkan Ohlsson Foundations, and from the Marie Curie Individual Intra‐European Fellowship within the EU FP6 Framework Program. The EPICure 2 cohort was funded by the Medical Research Council (G0401525). The EPIPAGE 2 cohort has been funded with support from the French Institute of Public Health Research/Institute of Public Health and its partners: the French Health Ministry, the National Institute of Health and Medical Research (INSERM), the National Institute of Cancer, and the National Solidarity Fund for Autonomy (CNSA); The National Research Agency, through the French EQUIPEX program of Investments in the Future (reference ANR‐11‐EQPX‐0038); the PREMUP Foundation; and the Foundation of France (reference 00050329). The funders had no role in study design, data collection, data analysis, data interpretation, decision to publish, or preparation of the manuscript.

## CONFLICT OF INTEREST STATEMENT

Andrei S. Morgan, Jennifer Zeitlin, Karin Källén, Elizabeth S. Draper, Stef van Buuren, Samantha Johnson, Valérie Benhammou, and Pierre‐Yves Ancel report grants from H2020/European Union during the conduct of this study; Mikael Norman reports grants from Stockholm County Council and Karolinksa Institutet (ALF 2020–0443) during the conduct of this study and personal fees from AbbVie and Chiesi Pharma AB; and Neil Marlow reports grants from the Medical Research Council during the conduct of the study and personal fees from Novartis, Takeda, and RSM Consulting outside the submitted work. The other authors declared no conflicts of interest.

## Supporting information


**TABLE S1:** Variables from EXPRESS, EPICure‐2 and EPIPAGE‐2 cohorts previously harmonized.
**TABLE S2:** Additional variables from EXPRESS, EPICure‐2 and EPIPAGE‐2 cohorts harmonized for this study.
**TABLE S3:** Characteristics of the variables used in imputation models.
**TABLE S4:** STROBE Checklist.
**TABLE S5:** Baseline characteristics – EXPRESS.
**TABLE S6:** Baseline characteristics – EPICure‐2.
**TABLE S7:** Baseline characteristics – EPIPAGE‐2.
**TABLE S8:** Outcomes – EXPRESS – Descriptive analysis.
**TABLE S9:** Outcomes – EPICure‐2 – Descriptive analysis.
**TABLE S10:** Outcomes – EPIPAGE‐2 – Descriptive analysis.

## Data Availability

The data that support the findings of this study are available from each cohort respectively. Restrictions may apply to the availability of these data, which were used under license for this study. Data are also available for analysis via the RECAP Preterm platform after appropriate permissions have been obtained: https://platform.recap‐preterm.eu.

## References

[1] NCD Risk Factor Collaboration (NCD‐RisC) . Trends in adult body‐mass index in 200 countries from 1975 to 2014: a pooled analysis of 1698 population‐based measurement studies with 19·2 million participants. Lancet. 2016;387(10026):1377‐1396. doi:10.1016/S0140-6736(16)30054-X 27115820 PMC7615134

[2] Heslehurst N , Ells LJ , Simpson H , Batterham A , Wilkinson J , Summerbell CD . Trends in maternal obesity incidence rates, demographic predictors, and health inequalities in 36 821 women over a 15‐year period. BJOG. 2007;114(2):187‐194. doi:10.1111/j.1471-0528.2006.01180.x 17305899

[3] Poston L , Caleyachetty R , Cnattingius S , et al. Preconceptional and maternal obesity: epidemiology and health consequences. Lancet Diabetes Endocrinol. 2016;4(12):1025‐1036. doi:10.1016/S2213-8587(16)30217-0 27743975

[4] Obesity among women of childbearing age: US, 1990–2020 . PeriStats | March of Dimes. Accessed June 16, 2022. https://www.marchofdimes.org/peristats/data?reg=99&top=17&stop=350&lev=1&slev=1&obj=8&cmp=99&eny=2020&sty=1990

[5] Euro‐Peristat Project . European Perinatal Health Report. Core indicators of the health and care of pregnant women and babies in Europe in 2015. November 2018. www.europeristat.com

[6] Black RE , Victora CG , Walker SP , et al. Maternal and child undernutrition and overweight in low‐income and middle‐income countries. Lancet. 2013;382(9890):427‐451. doi:10.1016/S0140-6736(13)60937-X 23746772

[7] Ehrenberg HM , Dierker L , Milluzzi C , Mercer BM . Low maternal weight, failure to thrive in pregnancy, and adverse pregnancy outcomes. Am J Obstet Gynecol. 2003;189(6):1726‐1730. doi:10.1016/S0002-9378(03)00860-3 14710105

[8] Catalano PM , Shankar K . Obesity and pregnancy: mechanisms of short term and long term adverse consequences for mother and child. BMJ. 2017;356:356. doi:10.1136/bmj.j1 PMC6888512 28179267

[9] Ramsay JE , Ferrell WR , Crawford L , Wallace AM , Greer IA , Sattar N . Maternal obesity is associated with dysregulation of metabolic, vascular, and inflammatory pathways. J Clin Endocrinol Metabol. 2002;87(9):4231‐4237. doi:10.1210/jc.2002-020311 12213876

[10] Basu S , Haghiac M , Surace P , et al. Pregravid obesity associates with increased maternal endotoxemia and metabolic inflammation. Obesity. 2011;19(3):476‐482. doi:10.1038/oby.2010.215 20930711 PMC3628602

[11] Shook LL , Kislal S , Edlow AG . Fetal brain and placental programming in maternal obesity: a review of human and animal model studies. Prenat Diagn. 2020;40(9):1126‐1137. doi:10.1002/pd.5724 32362000 PMC7606714

[12] Aune D , Saugstad OD , Henriksen T , Tonstad S . Maternal body mass index and the risk of fetal death, stillbirth, and infant death: a systematic review and meta‐analysis. JAMA. 2014;311(15):1536. doi:10.1001/jama.2014.2269 24737366

[13] Koch L . Effect of maternal obesity on neonatal outcomes. Nat Rev Endocrinol. 2013;9(8):439. doi:10.1038/nrendo.2013.126 23797821

[14] Li M , Fallin MD , Riley A , et al. The association of maternal obesity and diabetes with autism and other developmental disabilities. Pediatrics. 2016;137(2): e20152206. doi:10.1542/peds.2015-2206 26826214 PMC4732357

[15] McDonald SD , Han Z , Mulla S , Beyene J . Overweight and obesity in mothers and risk of preterm birth and low birth weight infants: systematic review and meta‐analyses. BMJ. 2010;341:c3428. doi:10.1136/bmj.c3428 20647282 PMC2907482

[16] Cnattingius S , Villamor E , Johansson S , et al. Maternal obesity and risk of preterm delivery. JAMA. 2013;309(22):2362‐2370. doi:10.1001/jama.2013.6295 23757084

[17] EXPRESS Group , Fellman V , Hellström‐Westas L , et al. One‐year survival of extremely preterm infants after active perinatal care in Sweden. JAMA. 2009;301(21):2225‐2233. doi:10.1001/jama.2009.771 19491184

[18] Costeloe KL , Hennessy EM , Haider S , Stacey F , Marlow N , Draper ES . Short term outcomes after extreme preterm birth in England: comparison of two birth cohorts in 1995 and 2006 (the EPICure studies). BMJ. 2012;345:e7976. doi:10.1136/bmj.e7976 23212881 PMC3514472

[19] Ancel PY , Goffinet F , Kuhn P , et al. Survival and morbidity of preterm children born at 22 through 34 weeks' gestation in France in 2011: results of the EPIPAGE‐2 cohort study. JAMA Pediatr. 2015;169(3):230‐238. doi:10.1001/jamapediatrics.2014.3351 25621457

[20] Morgan AS , Zeitlin J , Källén K , et al. Birth outcomes between 22 and 26 weeks' gestation in national population‐based cohorts from Sweden, England and France. Acta Paediatr. 2022;111(1):59‐75. doi:10.1111/apa.16084 34469604 PMC9291863

[21] Volpe JJ . Brain injury in premature infants: a complex amalgam of destructive and developmental disturbances. Lancet Neurol. 2009;8(1):110‐124. doi:10.1016/S1474-4422(08)70294-1 19081519 PMC2707149

[22] Jobe AH , Bancalari E . Bronchopulmonary dysplasia. Am J Respir Crit Care Med. 2001;163(7):1723‐1729. doi:10.1164/ajrccm.163.7.2011060 11401896

[23] International Committee for the Classification of Retinopathy of Prematurity. The International Classification of Retinopathy of Prematurity revisited. Arch Ophthalmol. 2005;123(7):991‐999. doi:10.1001/archopht.123.7.991 16009843

[24] Serenius F , Ewald U , Farooqi A , Holmgren PÅ , Håkansson S , Sedin G . Short‐term outcome after active perinatal management at 23‐25 weeks of gestation. A study from two Swedish tertiary care centres. Part 2: infant survival. Acta Paediatr. 2004;93(8):1081‐1089. doi:10.1111/j.1651-2227.2004.tb02721.x 15456200

[25] Papile LA , Burstein J , Koffler H . Incidence and evolution of subependymal and intraventricular hemorrhage: a study of infants with birth weights less than 1,500 gm. J Pediatr. 1978;92(4):529‐534. doi:10.1016/s0022-3476(78)80282-0 305471

[26] de Vries LS , Eken P , Dubowitz LM . The spectrum of leukomalacia using cranial ultrasound. Behav Brain Res. 1992;49(1):1‐6. doi:10.1016/s0166-4328(05)80189-5 1388792

[27] Bonet M , Smith LK , Pilkington H , Draper ES , Zeitlin J . Neighbourhood deprivation and very preterm birth in an English and French cohort. BMC Pregnancy Childbirth. 2013;13(1):97. doi:10.1186/1471-2393-13-97 23617598 PMC3640897

[28] Khalak R , Rijhsinghani A , McCallum SE . Impact of maternal obesity on very preterm infants. Obesity. 2017;25(5):945‐949. doi:10.1002/oby.21812 28332298

[29] Chawla S , Laptook AR , Smith EA , et al. In‐hospital mortality and morbidity among extremely preterm infants in relation to maternal body mass index. J Perinatol. 2021;41(5):1014‐1024. doi:10.1038/s41372-020-00847-0 33024258 PMC8021608

[30] Mitha A , Chen R , Johansson S , Razaz N , Cnattingius S . Maternal body mass index in early pregnancy and severe asphyxia‐related complications in preterm infants. Int J Epidemiol. 2020;49(5):1647‐1660. doi:10.1093/ije/dyaa088 32588048 PMC7746401

[31] Pai VV , Carmichael SL , Kan P , Leonard SA , Lee HC . Maternal body mass index and risk of intraventricular hemorrhage in preterm infants. Pediatr Res. 2018;83(6):1146‐1151. doi:10.1038/pr.2018.47 29624572

[32] Carmichael SL , Kan P , Gould JB , Stevenson DK , Shaw GM , Lee HC . Maternal prepregnancy body mass index and risk of bronchopulmonary dysplasia. Pediatr Res. 2017;82(1):8‐13. doi:10.1038/pr.2017.90 28399116

[33] Johansson S , Villamor E , Altman M , Bonamy AKE , Granath F , Cnattingius S . Maternal overweight and obesity in early pregnancy and risk of infant mortality: a population based cohort study in Sweden. BMJ. 2014;349:g6572. doi:10.1136/bmj.g6572 25467170 PMC4252825

[34] Moreau M , Remy M , Nusinovici S , et al. Neonatal and neurodevelopmental outcomes in preterm infants according to maternal body mass index: a prospective cohort study. PLoS One. 2019;14(12):e0225027. doi:10.1371/journal.pone.0225027 31805081 PMC6894768

[35] Villamor E , Norman M , Johansson S , Cnattingius S . Maternal obesity and risk of early‐onset neonatal bacterial sepsis: Nationwide cohort and sibling‐controlled studies. Clin Infect Dis. 2021;73(9):e2656‐e2664. doi:10.1093/cid/ciaa783 32770206 PMC8757322

[36] Jensen ET , van der Burg JW , O'Shea TM , et al. The relationship of maternal prepregnancy body mass index and pregnancy weight gain to neurocognitive function at age 10 years among children born extremely preterm. J Pediatr. 2017;187:50‐57.e3. doi:10.1016/j.jpeds.2017.02.064 28341527 PMC5533624

[37] van der Burg JW , Jensen ET , van de Bor M , et al. Maternal obesity and attention‐related symptoms in the preterm offspring. Early Hum Dev. 2017;115:9‐15. doi:10.1016/j.earlhumdev.2017.08.002 28822870 PMC6082429

[38] Morgan AS , Mendonça M , Thiele N , David AL . Management and outcomes of extreme preterm birth. BMJ. 2022;376:e055924. doi:10.1136/bmj-2021-055924 35012942 PMC8744861

[39] Morgan AS , Khoshnood B , Diguisto C , et al. Intensity of perinatal care for extremely preterm babies and outcomes at a higher gestational age: evidence from the EPIPAGE‐2 cohort study. BMC Pediatr. 2020;20(1):8. doi:10.1186/s12887-019-1856-1 31910799 PMC6945524

