# Supplementary material for: Impact of maternal prepregnancy body mass index on neonatal outcomes following extremely preterm birth
Source: Obesity (Silver Spring). 2025 Feb 6;33(3):599–611. doi: 10.1002/oby.24241 (PMC11897853; doi:10.1002/oby.24241)
Supplement: Supplementary file 1 — TABLE S1: Variables from EXPRESS, EPICure‐2 and EPIPAGE‐2 cohorts previously harmonized. TABLE S2: Additional variables from EXPRESS, EPICure‐2 and EPIPAGE‐2 cohorts harmonized for this study. TABLE S3: Characteristics of the variables used in imputation models. TABLE S4: STROBE Checklist. TABLE S5: Baseline characteristics – EXPRESS. TABLE S6: Baseline characteristics – EPICure‐2. TABLE S7: Baseline characteristics – EPIPAGE‐2. TABLE S8: Outcomes – EXPRESS – Descriptive analysis. TABLE S9: Outcomes – EPICure‐2 – Descriptive analysis. TABLE S10: Outcomes – EPIPAGE‐2 – Descriptive analysis. [file OBY-33-599-s001.docx]

**ONLINE SUPPORTING INFORMATION**

**Article title:** Impact of maternal pre-pregnancy body mass index on neonatal outcomes of extremely preterm infants

**Corresponding author:** Andrei Morgan

**Contact information:** [andrei.morgan@inserm.fr](mailto:andrei.morgan@inserm.fr)

Dr. Andrei Morgan, OPPaLE team, INSERM UMR 1153 (CRESS), Maternité Port Royal, 53 Avenue de l’Observatoire, Paris 75014, FRANCE

**OVERVIEW**

- Table S1: Variables from EXPRESS, EPICure-2 and EPIPAGE-2 cohorts previously harmonized – Page 2
- Table S2: Additional variables from EXPRESS, EPICure-2 and EPIPAGE-2 cohorts harmonised for this study – Page 3
- Details on Multiple Imputation – Page 4
- Table S3: Characteristics of the variables used in imputation models – Page 5
- Table S4: STROBE Checklist – Page 7
- Table S5: Baseline characteristics – EXPRESS – Page 10
- Table S6: Baseline characteristics – EPICure-2 – Page 11
- Table S7: Baseline characteristics – EPIPAGE-2 – Page 12
- Table S8: Outcomes – Express – Descriptive analysis – Page 13
- Table S9: Outcomes – EPICure-2 – Descriptive analysis – Page 14
- Table S10: Outcomes – EPIPAGE-2 – Descriptive analysis – Page 15

| **Table S1: Variables from EXPRESS, EPICure-2 and EPIPAGE-2 cohorts previously harmonised** | |
| --- | --- |
| **Variables used as coded** | **Variables transformed** |
| Nulliparity | Maternal age (years, continuous) recoded into 5 categories: <20, 20-24, 25-29, 30-34, 35 + |
| Pre-pregnancy diabetes |  |
| Pre-pregnancy high BP*^1^* |  |
| Pre-eclampsia | Birth weight (g, continuous) transformed in birth weight z-Score with Hadlock’s formula *^2^* (using the gestational age in weeks continuous variable) and recoded in 3 categories: < -2 SD*^1^*, between -2 and 2 SD*^1^* , > 2 SD*^1^* |
| Antenatal steroids (any) |  |
| Labour onset |  |
| Placental abruption |  |
| Level of neonatal unit at delivery hospital | Gestational age (weeks, continuous) recoded into 5 categories of completed weeks : 22, 23, 24, 25, 26 |
| Mode of delivery |  |
| Fetal sex |  |
| Congenital anomalies |  |
| Worst grade of IVH*^1^* |  |
| cPVL*^1^* |  |
| NEC*^1^* treated surgically |  |
| PDA*^1^* treated surgically |  |
| BPD*^1^* |  |
| Stage of ROP*^1^* |  |
| Treated ROP*^1^* |  |
| Survival at discharge |  |
| *^1^* Abbreviations: BP - Blood pressure ; SD – Standard deviation ; IVH - Intraventricular haemorrhage ; cPVL – Cystic periventricular leukomalacia ; NEC - Necrotizing enterocolitis ; PDA - Persistent ductus arteriosus ; BPD - Bronchopulmonary dysplasia ; ROP – Retinopathy of prematurity  *^2^* Hadlock’s formula: z-score = (birthweight-mean)/SD where mean = exp (0.578+0.332*gestational age-0.00354*gestational age^2) and SD = 0.127*(exp(0.578+0.332*gestational age-0.00354*gestational age^2)) | |

| **Table S2: Additional variables from EXPRESS, EPICure-2 and EPIPAGE-2 cohorts harmonised for this study** | | | | |
| --- | --- | --- | --- | --- |
| **Variables** | **EXPRESS** | **EPICure-2** | **EPIPAGE-2** | **Harmonised version** |
| Smoking | Maternal smoking in  pregnancy as reported at first antenatal visit: No, 1-9 cig, >10 cig per day | Maternal smoking at time of first booking: Yes / No | Any smoking during pregnancy (even for a short period): Yes / No | Yes / No |
| Gestational diabetes | Yes (Insulin dependent or not) / No | Yes / No | Yes / No | Yes / No |
| Antenatal transfer | Yes / No | Yes / No | Yes / No | Yes / No |
| Antenatal tocolytics | Yes (any) / No | Yes (any in the week prior to delivery) / No | Yes (tocolysis at hospital admission for delivery or last hospitalisation) / No | Yes / No |
| PPROM*^1^* | Rupture of membranes at  least one hour before the onset of contractions | Rupture of membranes > 24 hours before birth | Rupture of membranes > 12 hours before labour onset | Yes/No, according to each cohorts definition |
| Breastfeeding at discharge | Completely / Partly / No | Yes (any) / No | None / Mixed / Exclusive | Yes (exclusive or mixed) / No |
| Type of preterm birth | Composite variable created for the study using the « labour onset » and the « PPROM » variables and defining 3 categories: PPROM (if PPROM occurred whatever the onset of labour), spontaneous (if labour was spontaneous and PPROM didn’t occur or is not known) and medically indicated (if there was no labour or labour was induced and PPROM didn’t occur or is not known) | | | |
| At least one severe morbidity | Composite variable created for the study. Coded YES if one or more the severe morbidity was present and NO if none of them were present or information was missing: Severe neurological injury (severe IVH*^1^* grade III or IV using the Papille et al. classification and/or cPVL*^1^* according to de Vries et al.), surgical treatment for NEC*^1^*, surgical treatment for PDA*^1^*, severe BPD*^1^* (use of supplemental oxygen at 36 weeks’ postmenstrual age) and severe ROP*^1^* (stage 4 or 5 of the international classification and/or treated). | | | |
| Survival at discharge without any severe morbidity | Composite variable created for the study. Coded YES if the child survived to hospital discharge and the « at least one severe morbidity » variable is NO and NO if the child didn’t survive to hospital discharge and/or the « at least one severe morbidity » variable is YES. | | | |
| *^1^* Abbreviations: PPROM – Preterm premature rupture of membrane ; IVH - Intraventricular haemorrhage ; cPVL – Cystic periventricular leukomalacia ; NEC - Necrotizing enterocolitis ; PDA - Persistent ductus arteriosus ; BPD - Bronchopulmonary dysplasia ; ROP – Retinopathy of prematurity | | | | |

**Details on multiple imputation**

Multiple imputation by chained equations was used to account for missing data and implemented with the R-package "mice", following guidelines from Buuren and Groothuis-Oudshoorn (1). Their "seven step" method was used to choose the imputation settings:

1. We worked under a missing at random (MAR) assumption.
2. We used two imputation models: a logistic regression model for the binary variables and multinominal logistic regression model for categorical variables with more than 2 values. The only continuous variable ("gestational age at birth") had complete data and wasn’t imputed. Table S3 shows the amount of missing data and the imputation method used for each variable.
3. The prediction matrix was designed with the quickpred() function with a minimum proportion of usable cases of at least 0.25 and a minimum Pearson’s absolute correlation of at least 0.1. The prediction matrix was forced to take into account all variables included in the statistical models (confounders and outcomes). Constructed variables (i.e. that were functions of other, incomplete, variables) and ID variables were excluded from the prediction matrix.
4. We imputed constructed variables ("type of prematurity" and "any severe morbidity") using custom imputation methods to ensure that imputed values were concordant with related variables (e.g. "any severe morbidity", "bronchopulmonary-dysplasia" etc…).
5. We used the default visiting sequence (left to right).
6. We used 30 iterations to ensure models convergence.
7. Since some variables had high proportions of missing information, we created 60 imputed data sets.

Convergence was assessed for the different variables by plotting the mean and standard deviation, checking that they freely intermingled with each other. Plausibility of the imputation results was checked by plotting densities of both the observed and imputed values (using the densityplot() function from the lattice package). Analytical models were then applied to the multiple-imputed data, using Rubin’s rules (2) to combine estimates across the 60 data sets.

**References :**

1. Buuren S van, Groothuis-Oudshoorn K. **mice** : Multivariate Imputation by Chained Equations in *R*. J Stat Soft [Internet]. 2011 [cited 2022 Jun 21];45(3). Available from: http://www.jstatsoft.org/v45/i03/

2. Rubin DB. Frontmatter. In: Multiple Imputation for Nonresponse in Surveys [Internet]. John Wiley & Sons, Ltd; 1987 [cited 2022 Jun 21]. Available from: https://onlinelibrary.wiley.com/doi/abs/10.1002/9780470316696.fmatter

| **Table S3**. **Characteristics of the variables used in imputation models** | | | | | | | |
| --- | --- | --- | --- | --- | --- | --- | --- |
| **Variable name** | **Variable Type** | **N° of unique values** | **Model for data prediction** | **N° of missing values** (**N =** 4438) | **Missing rates, among:** | | |
|  |  |  |  |  | **All fetuses** | **Survivors at discharge** |  |
| Project ID*^1^* | Categorical | 4438 | No missing data | 0 | 0 | 0 |  |
| Maternal ID*^1^* | Categorical | 3870 | No missing data | 0 | 0 | 0 |  |
| High BP*^1^* | Binary | 2 | No missing data | 0 | 0 | 0 |  |
| Pre-Eclampsia | Binary | 2 | No missing data | 0 | 0 | 0 |  |
| Alive at maternal hospital admission | Binary | 1 | No missing data | 0 | 0 | 0 |  |
| Alive at birth | Binary | 2 | No missing data | 0 | 0 | 0 |  |
| Cohort | Categorical | 3 | No missing data | 0 | 0 | 0 |  |
| Multiple | Binary | 2 | No missing data | 0 | 0 | 0 |  |
| Alive at discharge | Binary | 2 | No missing data | 0 | 0 | 0 |  |
| Survivor at first follow-up | Binary | 2 | No missing data | 0 | 0 | 0 |  |
| Nulliparous | Binary | 2 | Logistic regression | 37 | 0.01 | 0.01 |  |
| Smoking | Binary | 2 | Logistic regression | 241 | 0.05 | 0.05 |  |
| Maternal age | Categorical | 7 | Multinominal logistic regression | 14 | 0 | 0 |  |
| Diabetes pre-pregnancy | Binary | 2 | Logistic regression | 67 | 0.02 | 0.02 |  |
| Gestational Diabetes | Binary | 2 | Logistic regression | 171 | 0.04 | 0.03 |  |
| Birth hospital level | Categorical | 3 | Multinominal logistic regression | 39 | 0.01 | 0.01 |  |
| Birth weight category | Categorical | 4 | Multinominal logistic regression | 66 | 0.01 | 0 |  |
| Sex | Binary | 2 | Logistic regression | 8 | 0 | 0 |  |
| Congenital anomaly | Binary | 2 | Logistic regression | 72 | 0.02 | 0.01 |  |
| Antenatal transfer | Binary | 2 | Logistic regression | 78 | 0.02 | 0.02 |  |
| Antenatal steroids | Binary | 2 | Logistic regression | 106 | 0.02 | 0.02 |  |
| Antenatal tocolysis | Binary | 2 | Logistic regression | 61 | 0.01 | 0.01 |  |
| Antenatal antibiotherapy | Binary | 2 | Logistic regression | 96 | 0.02 | 0.02 |  |
| PPROM*^1^* | Binary | 2 | Logistic regression | 354 | 0.08 | 0.11 |  |
| Placenta abruption | Binary | 2 | Logistic regression | 88 | 0.02 | 0.02 |  |
| Chorioamnionitis | Binary | 2 | Logistic regression | 341 | 0.08 | 0.06 |  |
| Labour type | Categorical | 3 | Multinominal logistic regression | 51 | 0.01 | 0.01 |  |
| Delivery presentation | Categorical | 3 | Multinominal logistic regression | 250 | 0.06 | 0.05 |  |
| Delivery type | Categorical | 3 | Multinominal logistic regression | 39 | 0.01 | 0 |  |
| Type of prematurity | Categorical | 3 | Custom | 380 | 0.09 | 0.12 |  |
| Admitted to NICU*^1^* | Binary | 2 | Logistic regression | 4 | 0 | 0 |  |
| BMI*^1^* category | Categorical | 6 | Multinominal logistic regression | 582 | 0.13 | 0.13 |  |
| NEC*^1^* surgery | Binary | 2 | Logistic regression | 749 | 0.17 | 0 |  |
| Intubated | Binary | 2 | Logistic regression | 767 | 0.17 | 0 |  |
| PDA *^1^*surgery | Binary | 2 | Logistic regression | 1373 | 0.31 | 0.01 |  |
| cPVL*^1^* | Binary | 2 | Logistic regression | 1424 | 0.32 | 0 |  |
| IVH*^1^* | Binary | 5 | Logistic regression | 1474 | 0.33 | 0 |  |
| BPD*^1^* | Binary | 3 | Logistic regression | 2334 | 0.53 | 0.05 |  |
| At least one severe morbidity | Binary | 2 | Custom | 2383 | 0.54 | 0.26 |  |
| Breastfeeding | Binary | 2 | Logistic regression | 2436 | 0.55 | 0.05 |  |
| ROP*^1^* Stages | Categorical | 6 | Multinominal logistic regression | 2455 | 0.55 | 0.1 |  |
| Motor dysfunction | Categorical | 4 | Multinominal logistic regression | 2783 | 0.63 | 0.21 |  |
| Vision impairment | Categorical | 3 | Multinominal logistic regression | 2988 | 0.67 | 0.31 |  |
| Hearing impairment | Categorical | 3 | Multinominal logistic regression | 2979 | 0.67 | 0.3 |  |
| ROP*^1^* treated | Binary | 2 | Multinominal logistic regression | 3108 | 0.70 | 0.41 |  |
| Gestational age at birth | Numeric |  | No missing data | 0 | 0 | 0 |  |
| *^1^* Abbreviations: ID – Identification ; BP - Blood pressure ; PPROM – Preterm premature rupture of membrane ; NICU – Neonatal intensive care unit ; BMI – Body mass index ; NEC - Necrotizing enterocolitis ; PDA - Persistent ductus arteriosus ; cPVL – Cystic periventricular leukomalacia ; IVH - Intraventricular haemorrhage ; BPD - Bronchopulmonary dysplasia ; ROP – Retinopathy of prematurity | | | | | | | |

|  |  | **Table S4: STROBE Checklist** |  |
| --- | --- | --- | --- |
| **Item** | **Item No** | **STROBE Recommendation** | **To be found in this article** |
| **Title and abstract** | 1 | (a) Indicate the study's design with a commonly used term in the title or the abstract | Abstract: Design section. |
|  |  | (b) Provide in the abstract an informative and balanced summary of what was done and what was found | Abstract: Main oucome and measure and Results sections. |
| **Introduction** |  |  |  |
| Background/ rationale | 2 | Explain the scientific background and rationale for the investigation being reported | Introduction (paragraphs 2, 3 and 4). |
| Objectives | 3 | State specific objectives, including any prespecified hypotheses | Introduction (paragraph 5). |
| **Methods** | | | |
| Study design | 4 | Present key elements of study design early in the paper | Methods (Data sources section). |
| Setting | 5 | Describe the setting, locations, and relevant dates, including periods of recruitment, exposure, follow-up, and data collection | Methods (Data sources section). |
| Participants | 6 | (a) Give the eligibility criteria, and the sources and methods of selection of participants. Describe methods of follow-up | Methods (Data sources and Study Population sections). |
|  |  | (b) For matched studies, give matching criteria and number of exposed and unexposed | N/A |
| Variables | 7 | Clearly define all outcomes, exposures, predictors, potential confounders, and effect modifiers. Give diagnostic criteria, if applicable | Methods (Harmonisation, Exposure, Outcomes and other variables sections) ; eFigure 1 ; eTable 1and 2 |
| Data sources/ measurement | 8* | For each variable of interest, give sources of data and details of methods of assessment (measurement). Describe comparability of assessment methods if there is more than one group. | Methods (Harmonisation, Exposure, Outcomes and other variables sections) ; eTable 1and 2 |
| Bias | 9 | Describe any efforts to address potential sources of bias | Methods (Statistical analysis section) ; eFigure 1 ; eTable 3 ; Details on multiple imputation |
| Study size | 10 | Explain how the study size was arrived at | Methods (Data sources and Study Population sections) and Results (Descriptive analysis section) |
| Quantitative variables | 11 | Explain how quantitative variables were handled in the analyses. If applicable, describe which groupings were chosen and why | N/A |
| Statistical methods | 12 | (a) Describe all statistical methods, including those used to control for confounding | Methods (Statistical analysis section). |
|  |  | (b) Describe any methods used to examine subgroups and interactions | Methods (Statistical analysis section). |
|  |  | (c) Explain how missing data were addressed | Methods (Statistical analysis section) ; eTable 3 ; Details on multiple imputation |
|  |  | (d) If applicable, explain how loss to follow-up was addressed | N/A |
|  |  | (e) Describe any sensitivity analyses | Methods (Statistical analysis section). |
| **Results** | | | |
| Participants | 13* | (a) Report numbers of individuals at each stage of study—eg numbers potentially eligible, examined for eligibility, confirmed eligible, included in the study, completing follow-up, and analysed | Results (Descriptive analysis section) ; Figure 1 |
|  |  | (b) Give reasons for non-participation at each stage | N/A |
|  |  | (c) Consider use of a flow diagram | Figure 1 |
| Descriptive data | 14* | (a) Give characteristics of study participants (eg demographic, clinical, social) and information on exposures and potential confounders | Results (Descriptive analysis section) ; Table 1 |
|  |  | (b) Indicate number of participants with missing data for each variable of interest | Table 1 |
|  |  | (c) Summarise follow-up time (eg, average and total amount) | N/A |
| Outcome data | 15* | Report numbers of outcome events or summary measures over time | Table 2 |
| Main results | 16 | (*a*) Give unadjusted estimates and, if applicable, confounder-adjusted estimates and their precision (eg, 95% confidence interval). Make clear which confounders were adjusted for and why they were included | Results (Primary outcome and secondary outcome sections) ; eFigure 1 ; Table 3 and 4 |
|  |  | (*b*) Report category boundaries when continuous variables were categorized | N/A |
|  |  | (*c*) If relevant, consider translating estimates of relative risk into absolute risk for a meaningful time period | N/A |
| Other analyses | 17 | Report other analyses done—eg analyses of subgroups and interactions, and sensitivity analyses | Results (Primary outcome section) ; Table 3 and 4 ; Data available on request. |
| **Discussion** | | | |
| Key results | 18 | Summarise key results with reference to study objectives | Discussion (Principal findings section) |
| Limitations | 19 | Discuss limitations of the study, taking into account sources of potential bias or imprecision. Discuss both direction and magnitude of any potential bias | Discussion (Strength and weaknesses) |
| Interpretation | 20 | Give a cautious overall interpretation of results considering objectives, limitations, multiplicity of analyses, results from similar studies, and other relevant evidence | Discussion (Comparison with literature section) |
| Generalisability | 21 | Discuss the generalisability (external validity) of the study results | Discussion (Implication section) |
| **Other information** | | | |
| Funding | 22 | Give the source of funding and the role of the funders for the present study and, if applicable, for the original study on which the present article is based | Fundings section |

*Give information separately for exposed and unexposed groups.

**Note:** An Explanation and Elaboration article discusses each checklist item and gives methodological background and published examples of transparent reporting. The STROBE checklist is best used in conjunction with this article (freely available on the Web sites of PLoS Medicine at http://www.plosmedicine.org/, Annals of Internal Medicine at http://www.annals.org/, and Epidemiology at http://www.epidem.com/). Information on the STROBE Initiative is available at http://www.strobe-statement.org.

| **Table S5: Baseline characteristics – Express – [ n/N (%)]** | | | | | | |
| --- | --- | --- | --- | --- | --- | --- |
|  | **Complete cases -** N = 534 | | | | **Missing BMI***^1^*  N = 66 | **Total**  N = 600 |
|  | **BMI***^1^* **<18.5**  N = 14 | **BMI***^1^* **18.5-24.9**  N = 293 | **BMI***^1^* **25-29.9**  N = 128 | **BMI***^1^* **>30**  N = 99 |  |  |
| 1. **Mother** | | | | | | |
| **Maternal age (years)** |  |  |  |  |  |  |
| <20 | 2/14 (14.3) | 14/291 (4.8) | 2/128 (1.6) | 4/98 (4.1) | 4/65 (6.2) | 26/596 (4.4) |
| 20-24 | 3/14 (21.4) | 34/291 (11.7) | 18/128 (14.1) | 7/98 (7.1) | 9/65 (13.8) | 71/596 (11.9) |
| 25-29 | 5/14 (35.7) | 68/291 (23.4) | 32/128 (25.0) | 25/98 (25.5) | 21/65 (32.3) | 151/596 (25.3) |
| 30-34 | 3/14 (21.4) | 96/291 (33.0) | 32/128 (25.0) | 31/98 (31.6) | 20/65 (30.8) | 182/596 (30.5) |
| 35 + | 1/14 (7.1) | 79/291 (27.1) | 44/128 (34.4) | 31/98 (31.6) | 11/65 (16.9) | 166/596 (27.9) |
| **Nulliparity** | 5/14 (35.7) | 139/293 (47.4) | 49/128 (38.3) | 32/99 (32.3) | 18/66 (27.3) | 243/600 (40.5) |
| **Smoking** | 4/13 (30.8) | 25/290 (8.6) | 19/122 (15.6) | 18/98 (18.4) | 6/32 (18.8) | 72/555 (13.0) |
| **Pre-pregnancy diabetes** | 0/11 (0.0) | 2/274 (0.7) | 0/123 (0.0) | 3/98 (3.1) | 1/60 (1.7) | 6/566 (1.1) |
| **Gestational diabetes** | 0/11 (0.0) | 2/273 (0.7) | 0/123 (0.0) | 0/98 (0.0) | 0/60 (0.0) | 2/565 (0.4) |
| **Pre-pregnancy high BP***^1^* | 0/14 (0.0) | 9/293 (3.1) | 7/128 (5.5) | 8/99 (8.1) | 0/66 (0.0) | 24/600 (4.0) |
| **Pre-eclampsia** | 1/14 (7.1) | 28/293 (9.6) | 21/128 (16.4) | 14/99 (14.1) | 8/66 (12.1) | 72/600 (12.0) |
| 1. **Obstetrics** | | | | | | |
| **Antenatal transfer** | 7/11 (63.6) | 153/265 (57.7) | 68/119 (57.1) | 60/94 (63.8) | 31/58 (53.4) | 319/547 (58.3) |
| **Antenatal steroids** | 11/12 (91.7) | 227/278 (81.7) | 104/123 (84.6) | 83/97 (85.6) | 44/59 (74.6) | 469/569 (82.4) |
| **Antenatal tocolytics** | 11/12 (91.7) | 158/279 (56.6) | 66/122 (54.1) | 52/96 (54.2) | 34/61 (55.7) | 321/570 (56.3) |
| **Type of prematurity** |  |  |  |  |  |  |
| PPROM*^1^* | 1/11 (9.1) | 46/233 (19.7) | 17/108 (15.7) | 13/81 (16.0) | 10/51 (19.6) | 87/484 (18.0) |
| Spontaneous | 8/11 (72.7) | 122/233 (52.4) | 59/108 (54.6) | 40/81 (49.4) | 26/51 (51.0) | 255/484 (52.7) |
| Medically indicated | 2/11 (18.2) | 65/233 (27.9) | 32/108 (29.6) | 28/81 (34.6) | 15/51 (29.4) | 142/484 (29.3) |
| **Placental abruption** | 0/11 (0.0) | 36/271 (13.3) | 17/122 (13.9) | 15/97 (15.5) | 10/59 (16.9) | 78/560 (13.9) |
| **Level of birth hospital** |  |  |  |  |  |  |
| 1 | 1/14 (7.1) | 5/290 (1.7) | 3/128 (2.3) | 2/98 (2.0) | 5/65 (7.7) | 16/595 (2.7) |
| 2 | 2/14 (14.3) | 50/290 (17.2) | 31/128 (24.2) | 19/98 (19.4) | 16/65 (24.6) | 118/595 (19.8) |
| 3 | 11/14 (78.6) | 235/290 (81.0) | 94/128 (73.4) | 77/98 (78.6) | 44/65 (67.7) | 461/595 (77.5) |
| **Mode of delivery** |  |  |  |  |  |  |
| Vaginal | 8/14 (57.1) | 160/293 (54.6) | 70/128 (54.7) | 47/99 (47.5) | 42/66 (63.6) | 327/600 (54.5) |
| Caesarean section | 6/14 (42.9) | 133/293 (45.4) | 58/128 (45.3) | 52/99 (52.5) | 24/66 (36.4) | 273/600 (45.5) |
| 1. **Infant** | | | | | | |
| **Gestational age (weeks)** |  |  |  |  |  |  |
| 22 | 1/14 (7.1) | 28/293 (9.6) | 14/128 (10.9) | 6/99 (6.1) | 10/66 (15.2) | 59/600 (9.8) |
| 23 | 0/14 (0.0) | 50/293 (17.1) | 19/128 (14.8) | 18/99 (18.2) | 15/66 (22.7) | 102/600 (17.0) |
| 24 | 1/14 (7.1) | 59/293 (20.1) | 25/128 (19.5) | 17/99 (17.2) | 12/66 (18.2) | 114/600 (19.0) |
| 25 | 9/14 (64.3) | 79/293 (27.0) | 31/128 (24.2) | 33/99 (33.3) | 15/66 (22.7) | 167/600 (27.8) |
| 26 | 3/14 (21.4) | 77/293 (26.3) | 39/128 (30.5) | 25/99 (25.3) | 14/66 (21.2) | 158/600 (26.3) |
| **Sex (Female)** | 8/14 (57.1) | 126/292 (43.2) | 55/128 (43.0) | 48/99 (48.5) | 30/66 (45.5) | 267/599 (44.6) |
| **Birth weight Z Score** *^2^* |  |  |  |  |  |  |
| < -2 SD*^1^* | 1/14 (7.1) | 31/287 (10.8) | 18/128 (14.1) | 19/99 (19.2) | 6/65 (9.2) | 75/593 (12.6) |
| Between -2 and 2 SD*^1^* | 12/14 (85.7) | 251/287 (87.5) | 109/128 (85.2) | 77/99 (77.8) | 57/65 (87.7) | 506/593 (85.3) |
| > 2 SD*^1^* | 1/14 (7.1) | 5/287 (1.7) | 1/128 (0.8) | 3/99 (3.0) | 2/65 (3.1) | 12/593 (2.0) |
| **Congenital anomalies** | 1/14 (7.1) | 28/293 (9.6) | 13/128 (10.2) | 14/99 (14.1) | 5/66 (7.6) | 61/600 (10.2) |
| *^1^* Abbreviations: BMI - Body mass index ; BP - Blood pressure ; PPROM – Preterm premature rupture of membranes ; SD – Standard deviation  *^2^* Calculated with Hadlock’s formula | | | | | | |

| **Table S6: Baseline characteristics – EPICure-2 – [ n/N (%)]** | | | | | | |
| --- | --- | --- | --- | --- | --- | --- |
|  | **Complete cases -** N = 1480 | | | | **Missing BMI***^1^*  N = 241 | **Total**  N = 1721 |
|  | **BMI***^1^* **<18.5**  N = 56 | **BMI***^1^* **18.5-24.9**  N = 657 | **BMI***^1^* **25-29.9**  N =443 | **BMI***^1^* **>30**  N = 324 |  |  |
| 1. **Mother** | | | | | | |
| **Maternal age (years)** |  |  |  |  |  |  |
| <20 | 9/56 (16.1) | 85/657 (12.9) | 28/443 (6.3) | 9/324 (2.8) | 26/237 (11.0) | 157/1,717 (9.1) |
| 20-24 | 21/56 (37.5) | 142/657 (21.6) | 80/443 (18.1) | 55/324 (17.0) | 46/237 (19.4) | 344/1,717 (20.0) |
| 25-29 | 14/56 (25.0) | 182/657 (27.7) | 127/443 (28.7) | 91/324 (28.1) | 53/237 (22.4) | 467/1,717 (27.2) |
| 30-34 | 8/56 (14.3) | 137/657 (20.9) | 108/443 (24.4) | 83/324 (25.6) | 52/237 (21.9) | 388/1,717 (22.6) |
| 35 + | 4/56 (7.1) | 111/657 (16.9) | 100/443 (22.6) | 86/324 (26.5) | 60/237 (25.3) | 361/1,717 (21.0) |
| **Nulliparity** | 25/56 (44.6) | 309/651 (47.5) | 231/439 (52.6) | 168/319 (52.7) | 125/235 (53.2) | 858/1,700 (50.5) |
| **Smoking** | 16/53 (30.2) | 190/632 (30.1) | 114/424 (26.9) | 48/313 (15.3) | 51/193 (26.4) | 419/1,615 (25.9) |
| **Pre-pregnancy diabetes** | 0/56 (0.0) | 4/657 (0.6) | 6/443 (1.4) | 7/324 (2.2) | 2/239 (0.8) | 19/1,719 (1.1) |
| **Gestational diabetes** | 0/56 (0.0) | 1/657 (0.2) | 6/443 (1.4) | 4/324 (1.2) | 1/239 (0.4) | 12/1,719 (0.7) |
| **Pre-pregnancy high BP***^1^* | 0/56 (0.0) | 8/657 (1.2) | 11/443 (2.5) | 26/324 (8.0) | 8/241 (3.3) | 53/1,721 (3.1) |
| **Pre-eclampsia** | 1/56 (1.8) | 38/657 (5.8) | 31/443 (7.0) | 32/324 (9.9) | 22/241 (9.1) | 124/1,721 (7.2) |
| 1. **Obstetrics** | | | | | | |
| **Antenatal transfer** | 11/56 (19.6) | 117/657 (17.8) | 77/443 (17.4) | 53/324 (16.4) | 76/241 (31.5) | 334/1,721 (19.4) |
| **Antenatal steroids** | 38/56 (67.9) | 475/656 (72.4) | 315/439 (71.8) | 222/324 (68.5) | 167/231 (72.3) | 1,217/1,706 (71.3) |
| **Antenatal tocolytics** | 12/55 (21.8) | 172/655 (26.3) | 107/441 (24.3) | 75/324 (23.1) | 60/234 (25.6) | 426/1,709 (24.9) |
| **Type of prematurity** |  |  |  |  |  |  |
| PPROM*^1^* | 16/55 (29.1) | 196/656 (29.9) | 136/442 (30.8) | 83/323 (25.7) | 67/229 (29.3) | 498/1,705 (29.2) |
| Spontaneous | 36/55 (65.5) | 400/656 (61.0) | 261/442 (59.0) | 193/323 (59.8) | 126/229 (55.0) | 1,016/1,705 (59.6) |
| Medically indicated | 3/55 (5.5) | 60/656 (9.1) | 45/442 (10.2) | 47/323 (14.6) | 36/229 (15.7) | 191/1,705 (11.2) |
| **Placental abruption** | 7/56 (12.5) | 63/656 (9.6) | 32/442 (7.2) | 19/322 (5.9) | 12/233 (5.2) | 133/1,709 (7.8) |
| **Level of birth hospital** |  |  |  |  |  |  |
| 1 | 3/53 (5.7) | 67/646 (10.4) | 41/434 (9.4) | 31/324 (9.6) | 5/236 (2.1) | 147/1,693 (8.7) |
| 2 | 18/53 (34.0) | 248/646 (38.4) | 141/434 (32.5) | 99/324 (30.6) | 77/236 (32.6) | 583/1,693 (34.4) |
| 3 | 32/53 (60.4) | 331/646 (51.2) | 252/434 (58.1) | 194/324 (59.9) | 154/236 (65.3) | 963/1,693 (56.9) |
| **Mode of delivery** |  |  |  |  |  |  |
| Vaginal | 48/56 (85.7) | 536/656 (81.7) | 362/443 (81.7) | 239/324 (73.8) | 175/237 (73.8) | 1,360/1,716 (79.3) |
| Caesarean section | 8/56 (14.3) | 120/656 (18.3) | 81/443 (18.3) | 85/324 (26.2) | 62/237 (26.2) | 356/1,716 (20.7) |
| 1. **Infant** | | | | | | |
| **Gestational age (weeks)** |  |  |  |  |  |  |
| 22 | 8/56 (14.3) | 72/657 (11.0) | 51/443 (11.5) | 51/324 (15.7) | 22/241 (9.1) | 204/1,721 (11.9) |
| 23 | 12/56 (21.4) | 109/657 (16.6) | 68/443 (15.3) | 51/324 (15.7) | 41/241 (17.0) | 281/1,721 (16.3) |
| 24 | 9/56 (16.1) | 149/657 (22.7) | 96/443 (21.7) | 69/324 (21.3) | 43/241 (17.8) | 366/1,721 (21.3) |
| 25 | 17/56 (30.4) | 173/657 (26.3) | 99/443 (22.3) | 80/324 (24.7) | 66/241 (27.4) | 435/1,721 (25.3) |
| 26 | 10/56 (17.9) | 154/657 (23.4) | 129/443 (29.1) | 73/324 (22.5) | 69/241 (28.6) | 435/1,721 (25.3) |
| **Sex (Female)** | 26/56 (46.4) | 298/656 (45.4) | 213/443 (48.1) | 153/324 (47.2) | 113/239 (47.3) | 803/1,718 (46.7) |
| **Birth weight Z Score** *^2^* |  |  |  |  |  |  |
| < -2 SD*^1^* | 6/55 (10.9) | 59/651 (9.1) | 49/441 (11.1) | 34/320 (10.6) | 22/238 (9.2) | 170/1,705 (10.0) |
| Between -2 and 2 SD*^1^* | 48/55 (87.3) | 582/651 (89.4) | 383/441 (86.8) | 283/320 (88.4) | 213/238 (89.5) | 1,509/1,705 (88.5) |
| > 2 SD*^1^* | 1/55 (1.8) | 10/651 (1.5) | 9/441 (2.0) | 3/320 (0.9) | 3/238 (1.3) | 26/1,705 (1.5) |
| **Congenital anomalies** | 1/55 (1.8) | 17/636 (2.7) | 7/426 (1.6) | 9/317 (2.8) | 4/230 (1.7) | 38/1,664 (2.3) |
| *^1^* Abbreviations: BMI - Body mass index ; BP - Blood pressure ; PPROM – Preterm premature rupture of membranes ; SD – Standard deviation  *^2^* Calculated with Hadlock’s formula | | | | | | |

| **Table S7: Baseline characteristics – EPIPAGE-2 – [ n/N (%)]** | | | | | | |
| --- | --- | --- | --- | --- | --- | --- |
|  | **Complete cases -** N = 797 | | | | **Missing BMI***^1^*  N = 138 | **Total**  N = 935 |
|  | **BMI***^1^* **<18.5**  N = 70 | **BMI***^1^* **18.5-24.9**  N = 446 | **BMI***^1^* **25-29.9**  N = 148 | **BMI***^1^* **>30**  N = 133 |  |  |
| 1. **Mother** | | | | | | |
| **Maternal age (years)** |  |  |  |  |  |  |
| <20 | 9/70 (12.9) | 24/445 (5.4) | 3/147 (2.0) | 1/133 (0.8) | 8/138 (5.8) | 45/933 (4.8) |
| 20-24 | 19/70 (27.1) | 90/445 (20.2) | 30/147 (20.4) | 21/133 (15.8) | 34/138 (24.6) | 194/933 (20.8) |
| 25-29 | 22/70 (31.4) | 149/445 (33.5) | 47/147 (32.0) | 36/133 (27.1) | 44/138 (31.9) | 298/933 (31.9) |
| 30-34 | 16/70 (22.9) | 99/445 (22.2) | 38/147 (25.9) | 42/133 (31.6) | 31/138 (22.5) | 226/933 (24.2) |
| 35 + | 4/70 (5.7) | 83/445 (18.7) | 29/147 (19.7) | 33/133 (24.8) | 21/138 (15.2) | 170/933 (18.2) |
| **Nulliparity** | 30/69 (43.5) | 180/444 (40.5) | 73/148 (49.3) | 54/130 (41.5) | 63/134 (47.0) | 400/925 (43.2) |
| **Smoking** | 23/68 (33.8) | 104/430 (24.2) | 33/146 (22.6) | 20/129 (15.5) | 15/113 (13.3) | 195/886 (22.0) |
| **Pre-pregnancy diabetes** | 0/70 (0.0) | 2/439 (0.5) | 0/147 (0.0) | 7/130 (5.4) | 1/128 (0.8) | 10/914 (1.1) |
| **Gestational diabetes** | 1/65 (1.5) | 5/404 (1.2) | 4/131 (3.1) | 14/120 (11.7) | 4/113 (3.5) | 28/833 (3.4) |
| **Pre-pregnancy high BP***^1^* | 2/70 (2.9) | 4/446 (0.9) | 5/148 (3.4) | 13/133 (9.8) | 4/138 (2.9) | 28/935 (3.0) |
| **Pre-eclampsia** | 2/70 (2.9) | 35/446 (7.8) | 15/148 (10.1) | 16/133 (12.0) | 9/138 (6.5) | 77/935 (8.2) |
| 1. **Obstetrics** | | | | | | |
| **Antenatal transfer** | 26/70 (37.1) | 169/440 (38.4) | 58/146 (39.7) | 46/132 (34.8) | 27/135 (20.0) | 326/923 (35.3) |
| **Antenatal steroids** | 37/66 (56.1) | 240/433 (55.4) | 84/147 (57.1) | 66/126 (52.4) | 41/123 (33.3) | 468/895 (52.3) |
| **Antenatal tocolytics** | 40/69 (58.0) | 241/442 (54.5) | 78/147 (53.1) | 71/131 (54.2) | 60/134 (44.8) | 490/923 (53.1) |
| **Type of prematurity** |  |  |  |  |  |  |
| PPROM*^1^* | 30/66 (45.5) | 169/430 (39.3) | 46/146 (31.5) | 39/130 (30.0) | 36/128 (28.1) | 320/900 (35.6) |
| Spontaneous | 27/66 (40.9) | 205/430 (47.7) | 74/146 (50.7) | 67/130 (51.5) | 75/128 (58.6) | 448/900 (49.8) |
| Medically indicated | 9/66 (13.6) | 56/430 (13.0) | 26/146 (17.8) | 24/130 (18.5) | 17/128 (13.3) | 132/900 (14.7) |
| **Placental abruption** | 4/69 (5.8) | 33/438 (7.5) | 5/146 (3.4) | 4/129 (3.1) | 3/131 (2.3) | 49/913 (5.4) |
| **Level of birth hospital** |  |  |  |  |  |  |
| 1 | 6/70 (8.6) | 23/446 (5.2) | 10/148 (6.8) | 8/133 (6.0) | 16/138 (11.6) | 63/935 (6.7) |
| 2 | 9/70 (12.9) | 95/446 (21.3) | 31/148 (20.9) | 26/133 (19.5) | 43/138 (31.2) | 204/935 (21.8) |
| 3 | 55/70 (78.6) | 328/446 (73.5) | 107/148 (72.3) | 99/133 (74.4) | 79/138 (57.2) | 668/935 (71.4) |
| **Mode of delivery** |  |  |  |  |  |  |
| Vaginal | 43/67 (64.2) | 306/436 (70.2) | 105/148 (70.9) | 97/128 (75.8) | 95/133 (71.4) | 646/912 (70.8) |
| Caesarean section | 24/67 (35.8) | 130/436 (29.8) | 43/148 (29.1) | 31/128 (24.2) | 38/133 (28.6) | 266/912 (29.2) |
| 1. **Infant** | | | | | | |
| **Gestational age (weeks)** |  |  |  |  |  |  |
| 22 | 9/70 (12.9) | 46/446 (10.3) | 20/148 (13.5) | 22/133 (16.5) | 21/138 (15.2) | 118/935 (12.6) |
| 23 | 6/70 (8.6) | 59/446 (13.2) | 17/148 (11.5) | 21/133 (15.8) | 28/138 (20.3) | 131/935 (14.0) |
| 24 | 15/70 (21.4) | 79/446 (17.7) | 31/148 (20.9) | 26/133 (19.5) | 26/138 (18.8) | 177/935 (18.9) |
| 25 | 16/70 (22.9) | 102/446 (22.9) | 36/148 (24.3) | 22/133 (16.5) | 26/138 (18.8) | 202/935 (21.6) |
| 26 | 24/70 (34.3) | 160/446 (35.9) | 44/148 (29.7) | 42/133 (31.6) | 37/138 (26.8) | 307/935 (32.8) |
| **Sex (Female)** | 39/70 (55.7) | 193/444 (43.5) | 77/148 (52.0) | 58/132 (43.9) | 55/138 (39.9) | 422/932 (45.3) |
| **Birth weight Z Score** *^2^* |  |  |  |  |  |  |
| < -2 SD*^1^* | 8/68 (11.8) | 47/441 (10.7) | 23/146 (15.8) | 20/131 (15.3) | 18/133 (13.5) | 116/919 (12.6) |
| Between -2 and 2 SD*^1^* | 60/68 (88.2) | 392/441 (88.9) | 121/146 (82.9) | 109/131 (83.2) | 113/133 (85.0) | 795/919 (86.5) |
| > 2 SD*^1^* | 0/68 (0.0) | 2/441 (0.5) | 2/146 (1.4) | 2/131 (1.5) | 2/133 (1.5) | 8/919 (0.9) |
| **Congenital anomalies** | 0/70 (0.0) | 11/446 (2.5) | 1/148 (0.7) | 0/133 (0.0) | 2/136 (1.5) | 14/933 (1.5) |
| *^1^* Abbreviations: BMI - Body mass index ; BP - Blood pressure ; PPROM – Preterm premature rupture of membranes ; SD – Standard deviation  *^2^* Calculated with Hadlock’s formula | | | | | | |

| **Table S8 : Outcomes – Express – Descriptive analysis – [ n/N (%)]** | | | | | | |
| --- | --- | --- | --- | --- | --- | --- |
|  | | | | | | |
|  | **Complete cases** | | | | **Missing BMI***^1^* | **Total** |
|  | **BMI***^1^* **<18.5** | **BMI***^1^* **18.5-24.9** | **BMI***^1^* **25-29.9** | **BMI***^1^* **>30** |  |  |
|  | | | | | | |
| **Fetuses alive at maternal admission to hospital** | **N = 14** | **N = 293** | **N = 128** | **N = 99** | **N = 66** | **N = 600** |
| **Survival at discharge without any major morbidity***^2^* | 8/14 (57.1) | 81/293 (27.6) | 47/128 (36.7) | 30/99 (30.3) | 18/66 (27.3) | 184/600 (30.7) |
| **Survival at discharge** | 12/14 (85.7) | 192/293 (65.5) | 90/128 (70.3) | 66/99 (66.7) | 39/66 (59.1) | 399/600 (66.5) |
|  | | | | | | |
| **Among survivors at discharge** | **N = 12** | **N = 192** | **N = 90** | **N = 66** | **N =39** | **N = 399** |
| **Any major morbidity***^2^* | 4/12 (33.3) | 111/192 (57.8) | 43/90 (47.8) | 36/66 (54.5) | 21/39 (53.8) | 215/399 (53.9) |
| **IVH***^1^* |  |  |  |  |  |  |
| 0 | 6/12 (50.0) | 118/190 (62.1) | 56/90 (62.2) | 45/66 (68.2) | 24/38 (63.2) | 249/396 (62.9) |
| 1 | 1/12 (8.3) | 34/190 (17.9) | 19/90 (21.1) | 9/66 (13.6) | 5/38 (13.2) | 68/396 (17.2) |
| 2 | 3/12 (25.0) | 19/190 (10.0) | 5/90 (5.6) | 6/66 (9.1) | 5/38 (13.2) | 38/396 (9.6) |
| 3 | 2/12 (16.7) | 10/190 (5.3) | 5/90 (5.6) | 5/66 (7.6) | 2/38 (5.3) | 24/396 (6.1) |
| 4 | 0/12 (0.0) | 9/190 (4.7) | 5/90 (5.6) | 1/66 (1.5) | 2/38 (5.3) | 17/396 (4.3) |
| **cPVL***^1^* | 0/12 (0.0) | 10/192 (5.2) | 4/90 (4.4) | 5/66 (7.6) | 2/39 (5.1) | 21/399 (5.3) |
| **NEC***^1^***: surgically treated** | 0/12 (0.0) | 5/191 (2.6) | 1/90 (1.1) | 4/66 (6.1) | 3/35 (8.6) | 13/394 (3.3) |
| **PDA***^1^***: surgically treated** | 2/12 (16.7) | 63/192 (32.8) | 25/90 (27.8) | 17/66 (25.8) | 8/39 (20.5) | 115/399 (28.8) |
| **BPD***^1^* |  |  |  |  |  |  |
| None/mild | 8/12 (66.7) | 50/175 (28.6) | 26/82 (31.7) | 24/63 (38.1) | 13/34 (38.2) | 121/366 (33.1) |
| Moderate | 4/12 (33.3) | 88/175 (50.3) | 36/82 (43.9) | 18/63 (28.6) | 14/34 (41.2) | 160/366 (43.7) |
| Severe | 0/12 (0.0) | 37/175 (21.1) | 20/82 (24.4) | 21/63 (33.3) | 7/34 (20.6) | 85/366 (23.2) |
| **ROP***^1^* **(Stages)** |  |  |  |  |  |  |
| 0 | 3/12 (25.0) | 45/192 (23.4) | 36/90 (40.0) | 20/66 (30.3) | 6/39 (15.4) | 110/399 (27.6) |
| 1 | 3/12 (25.0) | 23/192 (12.0) | 14/90 (15.6) | 8/66 (12.1) | 11/39 (28.2) | 59/399 (14.8) |
| 2 | 4/12 (33.3) | 50/192 (26.0) | 14/90 (15.6) | 18/66 (27.3) | 8/39 (20.5) | 94/399 (23.6) |
| 3 | 2/12 (16.7) | 72/192 (37.5) | 24/90 (26.7) | 20/66 (30.3) | 13/39 (33.3) | 131/399 (32.8) |
| 4 | 0/12 (0.0) | 1/192 (0.5) | 1/90 (1.1) | 0/66 (0.0) | 0/39 (0.0) | 2/399 (0.5) |
| 5 | 0/12 (0.0) | 1/192 (0.5) | 1/90 (1.1) | 0/66 (0.0) | 1/39 (2.6) | 3/399 (0.8) |
| **ROP***^1^* **(Treated)** | 2/2 (100.0) | 49/99 (49.5) | 20/38 (52.6) | 12/33 (36.4) | 8/18 (44.4) | 91/190 (47.9) |
| **Breastfeeding at discharge** | 7/11 (63.6) | 109/166 (65.7) | 43/86 (50.0) | 20/55 (36.4) | 23/34 (67.6) | 202/352 (57.4) |
| *^1^* Abbreviations: BMI - Body mass index ; IVH - Intra ventricular haemorrhage ; cPVL – Cystic periventricular leukomalacia ; NEC - Necrotizing enterocolitis ; ROP – Retinopathy of prematurity ; BPD - Bronchopulmonary dysplasia ; PDA - Persistent ductus arteriosus  *^2^* Major neonatal morbidity: Severe neurological injury (severe IVH grade III or IV using the Papille et al. classification and/or cPVL according to de Vries et al.), surgical treatment for NEC, surgical treatment for PDA, severe BPD (use of supplemental oxygen at 36 weeks’ postmenstrual age) and severe ROP (stage 4 or 5 of the international classification and/or treated). | | | | | | |

| **Table S9 : Outcomes – EPICure-2 – Descriptive analysis – [ n/N (%)]** | | | | | | |
| --- | --- | --- | --- | --- | --- | --- |
|  | | | | | | |
|  | **Complete cases** | | | | **Missing BMI***^1^* | **Total** |
|  | **BMI***^1^* **<18.5** | **BMI***^1^* **18.5-24.9** | **BMI***^1^* **25-29.9** | **BMI***^1^* **>30** |  |  |
|  | | | | | | |
| **Fetuses alive at maternal admission to hospital** | **N = 56** | **N = 657** | **N = 443** | **N = 324** | **N = 241** | **N = 1721** |
| **Survival at discharge without any major morbidity***^2^* | 8/56 (14.3) | 119/657 (18.1) | 80/443 (18.1) | 57/324 (17.6) | 48/241 (19.9) | 312/1,721 (18.1) |
| **Survival at discharge** | 24/56 (42.9) | 289/657 (44.0) | 208/443 (47.0) | 143/324 (44.1) | 125/241 (51.9) | 789/1,721 (45.8) |
|  | | | | | | |
| **Among survivors at discharge** | **N = 24** | **N = 289** | **N = 208** | **N = 143** | **N = 125** | **N = 789** |
| **Any major morbidity***^2^* | 16/24 (66.7) | 170/289 (58.8) | 128/208 (61.5) | 86/143 (60.1) | 77/125 (61.6) | 477/789 (60.5) |
| **IVH***^1^* |  |  |  |  |  |  |
| 0 | 10/24 (41.7) | 127/288 (44.1) | 97/206 (47.1) | 64/142 (45.1) | 65/125 (52.0) | 363/785 (46.2) |
| 1 | 3/24 (12.5) | 52/288 (18.1) | 34/206 (16.5) | 25/142 (17.6) | 20/125 (16.0) | 134/785 (17.1) |
| 2 | 3/24 (12.5) | 54/288 (18.8) | 38/206 (18.4) | 31/142 (21.8) | 19/125 (15.2) | 145/785 (18.5) |
| 3 | 3/24 (12.5) | 15/288 (5.2) | 14/206 (6.8) | 5/142 (3.5) | 7/125 (5.6) | 44/785 (5.6) |
| 4 | 5/24 (20.8) | 40/288 (13.9) | 23/206 (11.2) | 17/142 (12.0) | 14/125 (11.2) | 99/785 (12.6) |
| **cPVL***^1^* | 2/24 (8.3) | 12/288 (4.2) | 14/206 (6.8) | 8/142 (5.6) | 9/125 (7.2) | 45/785 (5.7) |
| **NEC***^1^***: surgically treated** | 2/24 (8.3) | 23/289 (8.0) | 14/208 (6.7) | 5/143 (3.5) | 12/125 (9.6) | 56/789 (7.1) |
| **PDA***^1^***: surgically treated** | 5/24 (20.8) | 46/286 (16.1) | 31/205 (15.1) | 21/142 (14.8) | 18/125 (14.4) | 121/782 (15.5) |
| **BPD***^1^* |  |  |  |  |  |  |
| None/mild | 9/24 (37.5) | 101/289 (34.9) | 65/207 (31.4) | 40/143 (28.0) | 42/125 (33.6) | 257/788 (32.6) |
| Moderate | 3/24 (12.5) | 76/289 (26.3) | 65/207 (31.4) | 42/143 (29.4) | 33/125 (26.4) | 219/788 (27.8) |
| Severe | 12/24 (50.0) | 112/289 (38.8) | 77/207 (37.2) | 61/143 (42.7) | 50/125 (40.0) | 312/788 (39.6) |
| **ROP***^1^* **(Stages)** |  |  |  |  |  |  |
| 0 | 13/23 (56.5) | 112/287 (39.0) | 73/207 (35.3) | 54/142 (38.0) | 52/124 (41.9) | 304/783 (38.8) |
| 1 | 0/23 (0.0) | 53/287 (18.5) | 40/207 (19.3) | 26/142 (18.3) | 29/124 (23.4) | 148/783 (18.9) |
| 2 | 7/23 (30.4) | 68/287 (23.7) | 52/207 (25.1) | 35/142 (24.6) | 19/124 (15.3) | 181/783 (23.1) |
| 3 | 3/23 (13.0) | 52/287 (18.1) | 42/207 (20.3) | 27/142 (19.0) | 23/124 (18.5) | 147/783 (18.8) |
| 4 | 0/23 (0.0) | 1/287 (0.3) | 0/207 (0.0) | 0/142 (0.0) | 1/124 (0.8) | 2/783 (0.3) |
| 5 | 0/23 (0.0) | 1/287 (0.3) | 0/207 (0.0) | 0/142 (0.0) | 0/124 (0.0) | 1/783 (0.1) |
| **ROP***^1^* **(Treated)** | 2/10 (20.0) | 43/175 (24.6) | 30/134 (22.4) | 23/88 (26.1) | 17/72 (23.6) | 115/479 (24.0) |
| **Breastfeeding at discharge** | 9/23 (39.1) | 130/289 (45.0) | 94/207 (45.4) | 60/142 (42.3) | 32/125 (25.6) | 325/786 (41.3) |
| *^1^* Abbreviations: BMI - Body mass index ; IVH - Intra ventricular haemorrhage ; cPVL – Cystic periventricular leukomalacia ; NEC - Necrotizing enterocolitis ; ROP – Retinopathy of prematurity ; BPD - Bronchopulmonary dysplasia ; PDA - Persistent ductus arteriosus  *^2^* Major neonatal morbidity: Severe neurological injury (severe IVH grade III or IV using the Papille et al. classification and/or cPVL according to de Vries et al.), surgical treatment for NEC, surgical treatment for PDA, severe BPD (use of supplemental oxygen at 36 weeks’ postmenstrual age) and severe ROP (stage 4 or 5 of the international classification and/or treated). | | | | | | |

| **Table S10 : Outcomes – EPIPAGE-2 – Descriptive analysis – [ n/N (%)]** | | | | | | |
| --- | --- | --- | --- | --- | --- | --- |
|  | | | | | | |
|  | **Complete cases** | | | | **Missing BMI***^1^* | **Total** |
|  | **BMI***^1^* **<18.5** | **BMI***^1^* **18.5-24.9** | **BMI***^1^* **25-29.9** | **BMI***^1^* **>30** |  |  |
|  | | | | | | |
| **Fetuses alive at maternal admission to hospital** | **N = 70** | **N = 446** | **N = 148** | **N = 133** | **N = 138** | **N = 935** |
| **Survival at discharge without any major morbidity***^2^* | 20/70 (28.6) | 109/446 (24.4) | 36/148 (24.3) | 21/133 (15.8) | 22/138 (15.9) | 208/935 (22.2) |
| **Survival at discharge** | 29/70 (41.4) | 196/446 (43.9) | 63/148 (42.6) | 46/133 (34.6) | 40/138 (29.0) | 374/935 (40.0) |
|  | | | | | | |
| **Among survivors at discharge** | **N = 29** | **N =196** | **N = 63** | **N = 46** | **N = 40** | **N = 374** |
| **Any major morbidity***^2^* | 9/29 (31.0) | 87/196 (44.4) | 27/63 (42.9) | 25/46 (54.3) | 18/40 (45.0) | 166/374 (44.4) |
| **IVH***^1^* |  |  |  |  |  |  |
| 0 | 18/29 (62.1) | 93/196 (47.4) | 38/63 (60.3) | 25/46 (54.3) | 19/40 (47.5) | 193/374 (51.6) |
| 1 | 1/29 (3.4) | 40/196 (20.4) | 11/63 (17.5) | 7/46 (15.2) | 7/40 (17.5) | 66/374 (17.6) |
| 2 | 8/29 (27.6) | 47/196 (24.0) | 12/63 (19.0) | 12/46 (26.1) | 8/40 (20.0) | 87/374 (23.3) |
| 3 | 1/29 (3.4) | 9/196 (4.6) | 1/63 (1.6) | 1/46 (2.2) | 5/40 (12.5) | 17/374 (4.5) |
| 4 | 1/29 (3.4) | 7/196 (3.6) | 1/63 (1.6) | 1/46 (2.2) | 1/40 (2.5) | 11/374 (2.9) |
| **cPVL***^1^* | 2/29 (6.9) | 7/196 (3.6) | 0/63 (0.0) | 1/46 (2.2) | 1/40 (2.5) | 11/374 (2.9) |
| **NEC***^1^***: surgically treated** | 1/29 (3.4) | 10/196 (5.1) | 2/63 (3.2) | 3/46 (6.5) | 1/40 (2.5) | 17/374 (4.5) |
| **PDA***^1^***: surgically treated** | 3/28 (10.7) | 39/191 (20.4) | 14/62 (22.6) | 10/41 (24.4) | 11/38 (28.9) | 77/360 (21.4) |
| **BPD***^1^* |  |  |  |  |  |  |
| None/mild | 15/25 (60.0) | 119/178 (66.9) | 32/53 (60.4) | 20/39 (51.3) | 24/37 (64.9) | 210/332 (63.3) |
| Moderate | 6/25 (24.0) | 16/178 (9.0) | 5/53 (9.4) | 8/39 (20.5) | 6/37 (16.2) | 41/332 (12.3) |
| Severe | 4/25 (16.0) | 43/178 (24.2) | 16/53 (30.2) | 11/39 (28.2) | 7/37 (18.9) | 81/332 (24.4) |
| **ROP***^1^* **(Stages)** |  |  |  |  |  |  |
| 0 | 12/19 (63.2) | 70/123 (56.9) | 21/38 (55.3) | 13/34 (38.2) | 10/22 (45.5) | 126/236 (53.4) |
| 1 | 4/19 (21.1) | 26/123 (21.1) | 8/38 (21.1) | 11/34 (32.4) | 5/22 (22.7) | 54/236 (22.9) |
| 2 | 2/19 (10.5) | 22/123 (17.9) | 7/38 (18.4) | 7/34 (20.6) | 4/22 (18.2) | 42/236 (17.8) |
| 3 | 1/19 (5.3) | 5/123 (4.1) | 1/38 (2.6) | 3/34 (8.8) | 3/22 (13.6) | 13/236 (5.5) |
| 4 | 0/19 (0.0) | 0/123 (0.0) | 1/38 (2.6) | 0/34 (0.0) | 0/22 (0.0) | 1/236 (0.4) |
| 5 | 0/19 (0.0) | 0/123 (0.0) | 0/38 (0.0) | 0/34 (0.0) | 0/22 (0.0) | 0/236 (0.0) |
| **ROP***^1^* **(Treated)** | 0/14 (0.0) | 5/119 (4.2) | 4/35 (11.4) | 1/32 (3.1) | 1/22 (4.5) | 11/222 (5.0) |
| **Breastfeeding at discharge** | 8/22 (36.4) | 73/186 (39.2) | 21/61 (34.4) | 11/44 (25.0) | 17/36 (47.2) | 130/349 (37.2) |
| *^1^* Abbreviations: BMI - Body mass index ; IVH - Intra ventricular haemorrhage ; cPVL – Cystic periventricular leukomalacia ; NEC - Necrotizing enterocolitis ; ROP – Retinopathy of prematurity ; BPD - Bronchopulmonary dysplasia ; PDA - Persistent ductus arteriosus  *^2^* Major neonatal morbidity: Severe neurological injury (severe IVH grade III or IV using the Papille et al. classification and/or cPVL according to de Vries et al.), surgical treatment for NEC, surgical treatment for PDA, severe BPD (use of supplemental oxygen at 36 weeks’ postmenstrual age) and severe ROP (stage 4 or 5 of the international classification and/or treated). | | | | | | |
